# Male care and life history traits in mammals

**DOI:** 10.1038/ncomms11854

**Published:** 2016-06-14

**Authors:** Hannah E. R. West, Isabella Capellini

**Affiliations:** 1 School of Biological, Biomedical and Environmental Sciences University of Hull Cottingham Road, Hull HU6 7RX, UK

## Abstract

Male care has energetic and opportunity costs, and is more likely to evolve when males gain greater certainty of paternity or when future mating opportunities are scarce. However, little is known about the substantial benefits that males may provide to females and offspring. Using phylogenetic comparative methods and a sample of over 500 mammalian species, we show that mammals in which males carry the offspring have shorter lactation periods, which leads to more frequent breeding events. Provisioning the female is associated with larger litters and shorter lactation. Offspring of species with male care have similar weaning mass to those without despite being supported by a shorter lactation period, implying that they grow faster. We propose that males provide an energetic contribution during the most expensive time of female reproduction, lactation, and that different male care behaviours increase female fecundity, which in turn helps males offset the costs of caring.

## Abstract (web summary)

Males help care for offspring in about 10% of mammal species. Here, West and Capellini perform phylogenetic comparative analyses on a sample of 529 mammal species and find that male care is associated with shorter lactation periods by females, larger litters of offspring, and more frequent breeding events.

## Graphical abstract

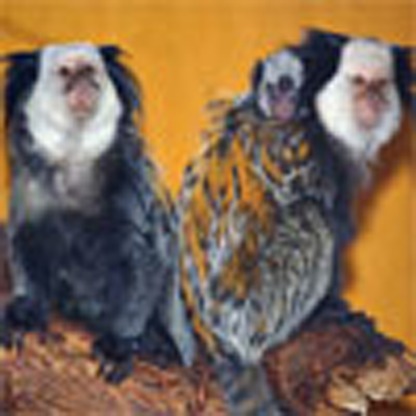

Parental care is any parental behaviour that benefits the offspring, frequently at the cost of survival or further mating opportunities for the carers^1^; therefore it should evolve when the carers' benefits outweigh the costs^2^. Parents provide no direct care in most species, yet females, males or both parents of different species across many lineages exhibit a great diversity of care behaviours^3^,^4^. The documented costs of male care in both vertebrates and invertebrates include increased risk of predation, parasitism or infection^5^,^6^,^7^,^8^,^9^, reduced mobility and foraging time^5^, leading to loss of body mass and condition^10^,^11^,^12^, loss of potential mating opportunities^13^, and in some species reduced survival, for example, (ref. 14). Given these costs, why do males care? Theoretical models and empirical studies show that males may trade-off costly care for a greater certainty or degree of paternity, reducing the level of care when female promiscuity is high^3^,^15^,^16^,^17^. Alternatively, when future mating opportunities are scarce, males might do better to care for their current offspring, regardless of paternity levels^18^. Most studies on the evolution of male care focus on the direct costs and benefits for the male, such as increased certainty of paternity, and the evolutionary relationship between male care and mating system^3^,^17^,^19^,^20^,^21^,^22^. While males invest a considerable amount of time and energy in caring, whether and how this leads to possible benefits to females and offspring is much less well understood^2^, particularly in species where females already care for the offspring (that is, biparental care). Quantifying these benefits, such as increased offspring survival and growth rates or female fecundity, is important because they could in turn increase the male's inclusive fitness and lead to evolutionary feedback between male care and life history traits^3^. Here, we investigate hypotheses that relate life history traits and male care at a large comparative scale in mammals, a taxon with obligatory female care and in which male care is also present in ∼10% of species^2^,^19^.

By providing an energetic contribution towards offspring rearing through costly care, such as provisioning dependent offspring or carrying heavy offspring, care by helpers, including the male, may allow females to redirect more resources into reproduction and in turn increase female reproductive success and/or offspring growth rates^2^,^20^,^23^. Males and additional carers may also enable females to spend more time foraging and gain more resources for current or future offspring^2^. The hypothesis that care by other individuals allows females to increase their reproductive output has been proposed mostly in the context of allocare (care by either the male or other individuals) for species with female care, such as birds and mammals (‘load-lightening' hypothesis^24^; see also Woodroffe and Vincent for male care^2^). Support for this hypothesis in relation to male care specifically is found in burying beetles (*Nicrophorus* sp.), where caring males help provisioning the offspring, and allow females to reduce their parental effort in the current brood and greatly increase their future brood mass^25^. Furthermore, female California mice (*Peromyscus californicus*) wean more offspring per reproductive bout and reproduce more frequently when males care^26^,^27^, indicating that females cannot meet the energetic demands of rearing more numerous and larger litters alone. Thus, it appears that the presence of male care has a ‘load-lightening' effect similar to that observed in species, such as meerkats (*Suricata suricatta*)^28^ and grey crowned babblers (*Pomatostomus temporalis*)^29^, where females helped by other carers can substantially increase their reproductive output^24^. However, whether male care is consistently associated with higher female fecundity across species is still poorly understood as the majority of studies addressing this question focus only on a few model species.

Large-scale comparative approaches are well suited to unravel the generality of patterns and processes^30^, but most comparative work on male care concerns primarily its evolutionary relationship with mating systems, for example, (refs 19, 20). The few comparative studies that test, at least partially, the hypothesis that male care associates with female fecundity focus on mammals. These studies find that litters are larger in species where females are helped by alloparents (males and/or other individuals)^31^,^32^, and that breeding frequency is higher in socially monogamous mammals in which males provision or carry the offspring^19^, a result also found in primates with allocare^31^. However, it is unclear whether a larger litter associates specifically with male care or care by other individuals, as previous studies do not separate care according to the identity of the carer, although benefits and costs of care may differ between the male and alloparents. Likewise, it is important to identify at which stage of reproduction male care is more likely to associate with higher female fecundity. Lactation is the most energetically demanding period of reproduction for a female mammal, with daily energy expenditure increasing by up to four times^33^,^34^,^35^. Male care may thus allow females to gain or save energy that can be (re)invested in more or better quality milk^20^,^23^, which in turn may result in a shorter lactation^2^,^23^ and lead to shorter interbirth intervals; if so, the documented association between frequency of breeding and male care^19^,^31^ is mediated by a reduction in the duration of lactation^2^. Alternatively, by caring for the offspring post-weaning, males may allow females to invest more time foraging, regain body condition more quickly and mate sooner, regardless of the duration of lactation^27^. Discriminating between these scenarios and identifying the relevant male care behaviour at a given stage of reproduction is fundamental because it helps to pinpoint the mechanism that underlies the evolutionary associations between male care and life history traits, and the possible evolutionary feedback between them.

Regardless of whether a higher frequency of breeding is achieved through male care post-weaning or by enabling females to wean the offspring sooner, higher female reproductive rates benefit the male only if he mates with the same female over more than one breeding event. This appears to be the case in mammals as recent comparative studies conclude that the evolution of social monogamy precedes the evolution of male care and is evolutionarily associated with it^19^,^20^. This evolutionary relationship may be especially relevant in long-lived species, as greater female fecundity over a longer lifespan could further help compensate for the loss of potential mating opportunities that should be experienced by monogamous caring males. Whether longer-lived species are more likely to exhibit male care is, however, unknown.

Like the care by other helpers, male care may also benefit the offspring by enhancing their survival to independence through protection against predators and/or by increasing offspring growth rates or size at independence^2^,^36^. Consistent with this hypothesis, zebra finch offspring (*Taeniopigia guttata*) have faster growth rate^37^ and snow bunting fledglings (*Plectrophenax nivalis*) are larger^38^ when raised by two parents than by one parent alone. Moreover, a non-phylogenetic study in carnivores finds that females have greater milk energetic output and offspring have higher growth rates in species where males or other individuals provision females and offspring^23^. However, we still do not know to what extent male care associates with greater offspring growth rates across mammals, and whether this leads to heavier offspring, which enjoy greater survival.

Although comparative studies cannot rule out the possibility that male care evolves in species where female fecundity or offspring growth are higher, the limited available experimental and field evidence in mammals and other organisms suggests that the absence of males caring for the offspring can have detrimental effects on both female fecundity, for example, (refs 25, 27) and offspring survival^39^,^40^,^41^,^42^. Yet, we currently lack a clear and comprehensive picture of how life history traits associate specifically with male care in any animal taxon at a large comparative scale, which is necessary to help direct future efforts aimed at disentangling cause and effect of the evolution of male care. Furthermore, previous comparative studies often analyze male care together with care by other individuals, but to what extent male care and care by others exhibit the same costs and benefits, and associations with life history traits, is unknown. In addition, previous comparative work considers only a subset of all male care behaviours under the assumption that some are more costly (for example, carrying and provisioning, most common in primates and carnivores)^19^,^31^. However, behaviours often regarded as less expensive, such as grooming and huddling with the offspring (most frequent in rodents), may entail substantial fitness costs for the male^10^,^11^,^43^,^44^, while allowing females to forage for longer periods and gain more resources for reproduction. Thus, identifying whose care—by the male or by other helpers—and which specific behaviour associates with life history traits is fundamental to understanding how and why male care evolves, as the benefits and costs of care are likely to vary in relation to the identity of the carer and the behaviour performed. Finally, assessing whether specific life history traits are evolutionary associated with male care also requires that the correlated evolution between life history traits is accounted for, as ignoring it may lead to misleading conclusions, as shown in (refs 45, 46).

Here, we compile the largest and most detailed dataset of male care behavior in mammals until now, and test the hypothesis that increased female fecundity and offspring fitness related traits are associated with male care using phylogenetic comparative methods. From this hypothesis, we test the predictions that species with male care exhibit (i) shorter lactation and/or gestation, (ii) more frequent and/or larger litters and (iii) larger neonates and/or weanlings. We consider both a broad definition of male care and each of the most frequently observed male care behaviours in mammals; provisioning the offspring, carrying, huddling and grooming. We also investigate provisioning reproducing females by the male, as this behaviour could indirectly benefit the offspring as well as the female. Using phylogenetic generalized least squares models (PGLS) to account for species' shared ancestry^47^,^48^, we build multi-predictor models where the dependent continuous variable is a life history trait of interest and male care is a binary independent variable. After accounting for allometry, the correlated evolution between life history traits^49^ and other potential confounding variables, we demonstrate that fecundity is higher in species with male care, but the way this is achieved is complex and varies across orders and the nature of male care. Litters are larger in species in which males provision reproducing females, especially carnivores, while frequency of breeding is higher in species with carrying, mostly primates, due to a reduction in lactation time. Lactation time is also shorter in carnivores where males provision reproducing females.

## Results

### Results across all mammals

**Figure 1:**
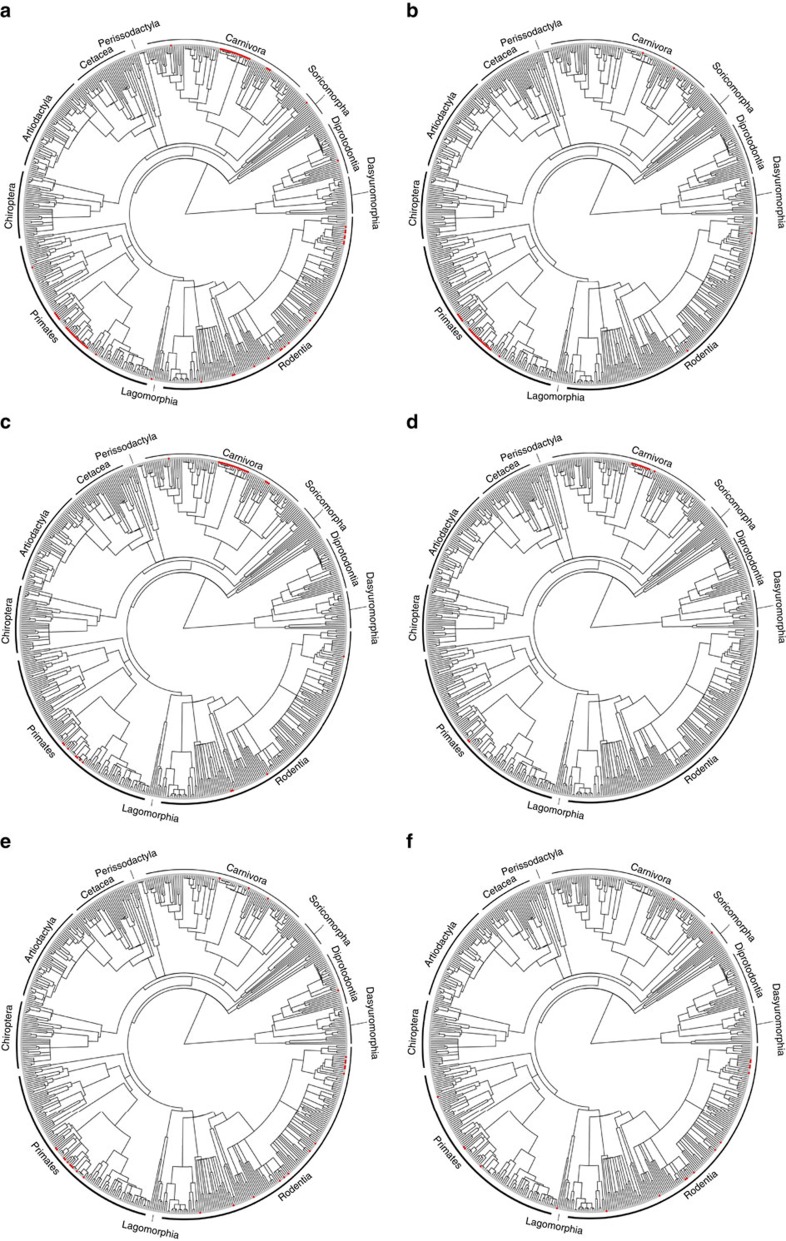
Distribution of male care behaviours across the mammal phylogeny. (**a**) Male care (any behaviour, red dots) in mammals (65 species with and 464 species without any form of male care). Species with biparental care (red dots) are indicated as follows: species with (**b**) carrying (*n*=27); (**c**) provisioning offspring (*n*=31); (**d**) provisioning reproducing females (*n*=15); (**e**) grooming (*n*=28); and (**f**) huddling with the offspring (*n*=19). In all panels, grey dots represent species without male care.

**Figure 2:**
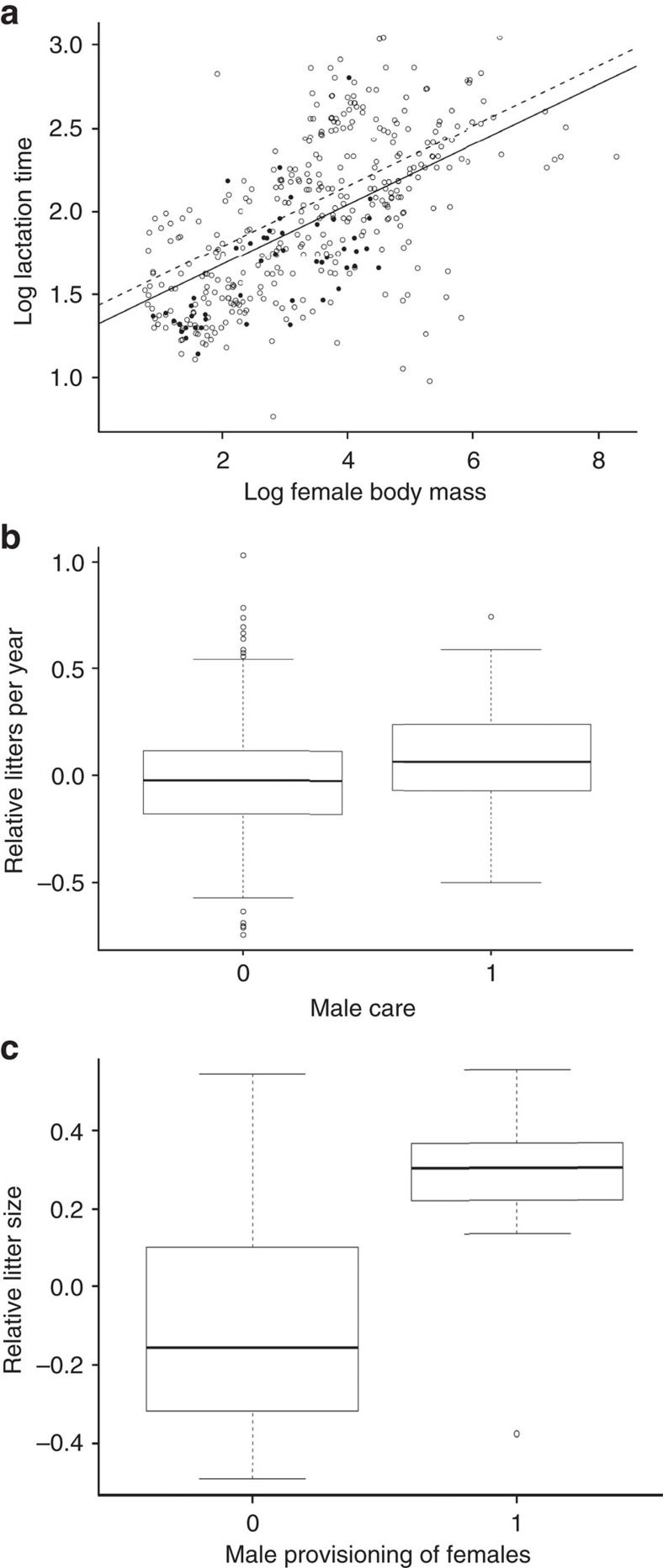
Male care and female life history traits across all mammals. (**a**) For a given female mass, lactation time is shorter in species with male care (filled circles) than species without it (open circles) (PGLS in Table 1: *n*=390). Best fitting line for species with male care in solid black, dashed line for species without male care. (**b**) The relative number of litters per year, after accounting for gestation time, lactation time and female mass, is higher in species with male care (coded as 1) than species without (coded as 0) (PGLS in Table 1: *n*=370). (**c**) Relative litter size, after accounting for female body mass, is larger in species where males provision reproducing females (coded as 1) than species without (coded as 0) (PGLS in Table 1: *n*=448). Figures in (**b**,**c**) report the median with upper and lower quartiles (boxes) and 95% confidence intervals (whiskers) of the residuals of litters per year (**b**) and litter size (**c**) computed from the reduced models in Table 1. All continuous data are log_10_-transformed.

Our analysis across 529 mammals with and without male care (Fig. 1) shows that lactation time is significantly shorter in species with male care (Fig. 2a), while accounting for allometry and gestation time (reduced model^50^ in Table 1), but is unrelated to all other predictors (full model 1 in Supplementary Table 1; likelihood ratio test for full versus reduced model: LR_3_=1.9, *P*=0.585). The amount of variance explained by the reduced model with male care increases by 2% relative to a model without it (LR_1_=6.1, *P*=0.013). When investigating individual behaviours, lactation time is shorter specifically in species where males carry the offspring while provisioning the female approaches significance (Table 1), but no other male care behaviour and no other predictor associates with lactation time (model 2 in Supplementary Table 1; full versus reduced model: LR_6_=2.6 *P*=0.857).

Gestation time is not associated with male care, after accounting for allometry and lactation time across all mammals (models 1 and 2, Supplementary Table 2), individual male care behaviours (model 3, Supplementary Table 2). However, species with care by other helpers have a significantly longer gestation than species without (model 2, Supplementary Table 2).

Frequency of breeding is higher in mammals with male care (Fig. 2b), with female body mass, care by helpers, lactation and gestation time being the only other predictors retained in the reduced model (Table 1; reduced model versus full model 1 in Supplementary Table 3: LR_2_=0.8, *P*=0.664). The reduced model with male care explains an additonal 1% of variance compared to a model without it (LR_1_=4.6, *P*=0.032). Among all care behaviours, grooming is the only significant predictor of litters per year, while accounting for allometry, the duration of maternal investment and care by helpers (Table 1, model 2 in Supplementary Table 3; full versus reduced model: LR_6_=4.66 *P*=0.588). The lack of a significant association between litters per year and carrying or provisioning females across mammals may reflect the fact that lactation is shorter in species exhibiting these behaviours (see above), and so most of the variance in litters per year, which could be explained by these behaviours, is likely explained by lactation time when the latter is included in the model. To investigate this possibility further, we repeat the analysis with all behaviours but excluding the duration of maternal investment, and find that breeding frequency is higher in species with carrying (Table 1, model 3 in Supplementary Table 3).

Although there is no significant relationship between litter size and male care (any behaviour) across all mammals (models 1 and 2 in Supplementary Table 4), litters are larger in species in which males provision reproducing females (Fig. 2c; Table 1, reduced model versus full model 3 in Supplementary Table 4: LR_6_=4.1, *P*=0.661). The reduced model with provisioning females and body size (Table 1) explains an additional 5% of variance in litter size than a model without it (LR_1_=24.3, *P*<0.001).

Mass at birth and mass gain from birth to weaning are unrelated to male care and individual male care behaviours across all mammals (Supplementary Table 5). Maximum lifespan is unrelated to male care or any individual male care behaviour, after accounting for the duration of lactation and gestation, and the number of litters per year (Supplementary Table 6).

Finally, including litter size as an additional predictor of lactation time and litters per year does not alter our results since litter size is not a significant predictor of lactation or litters per year (Supplementary Table 7).

### Order-specific results

Within individual orders with sufficient sample sizes for care behaviours (Supplementary Note 1), the duration of lactation is reduced in primates with carrying and in carnivores where males provision reproducing females, but it is unrelated to male care behaviours most common in rodents, namely huddling and grooming (Table 2; Supplementary Table 8). Gestation time is not associated with male care, after accounting for allometry and lactation time, within orders (Supplementary Table 9). Frequency of breeding is higher in primates with carrying due to a reduction in lactation time (Table 2; Supplementary Tables 8 and 10), but litters per year is unrelated to any care behaviour in carnivores and rodents (Supplementary Table 10). Litter size is unrelated to male care in primates, while provisioning reproducing females is associated with larger litters in carnivores (Table 2; Supplementary Table 11). Litter size is larger in socially monogamous rodents but unrelated to male care behaviours in this order (Supplementary Table 11). Mass at birth and mass gain from birth to weaning are unrelated to individual male care behaviours within each order where sample sizes are sufficiently large for analysis (Supplementary Tables 12 and 13). However, neonates are larger in carnivores with care by helpers and smaller in socially monogamous rodents (Supplementary Table 12). Sample sizes are too small to investigate the associations between male care and longevity within orders, and post-natal body mass gain in primates and carnivores (Supplementary Note 1).

## Discussion

Male care should evolve when the benefits of caring outweigh the costs to males' inclusive fitness. Potential benefits of male care include increased female fecundity and/or offspring fitness, which in turn provide fitness benefits to the caring male^2^,^19^,^20^,^23^. Thus, species with male care are expected to have more frequent and larger litters, shorter durations of maternal investment and heavier or faster growing offspring^2^,^23^. In support of this hypothesis, our comparative analysis reveals that male care is associated with increased female fecundity but differently across behaviours (Table 3). Specifically, litters are larger in species, mostly carnivores, where males provision reproducing females. Instead a reduction in lactation time in species with carrying, mostly but not exclusively primates, increases the frequency of breeding. Lactation is also shorter among carnivores, where males provision the females. Paternal care is however unrelated to prenatal maternal investment and offspring size at birth and weaning, suggesting that offspring grow faster postnatally but do not achieve a larger size in species with carrying and provisioning. Finally, longer-lived species are not more likely to exhibit male care. Taken together these results suggest that male care benefits both parents through increased female productivity, and that greater fecundity, but not a longer lifespan, helps maintaining the evolutionary association between social monogamy and male care.

By undertaking costly care or by caring for the offspring while the female forages for longer, males may help females meet the high energetic costs of lactation and allow them to invest more energy in milk production^20^,^23^. Consistent with this hypothesis, lactation time is shorter in mammals with male care; specifically with carrying the offspring or provisioning the females, behaviours most common in primates and carnivores, respectively. Conversely, lactation time is unrelated to huddling and grooming, suggesting that, overall, these male care behaviours may not help females change activity budgets sufficiently to enhance milk energy output. Although male care explains a small additional amount of variance, the reduction in the duration of lactation in species with male care can be substantial. For example, we estimate from a simple PGLS model including only female body mass and male care that, for a 10 kg mammal with biparental care, lactation is 31 days shorter (104 days) than that of a species of the same size without male care (135 days). While a previous study shows that allocare (including male care) in primates is associated with shorter lactation^31^, our analysis reveals that this effect is specifically linked to the presence of caring males, but not other helpers (Table 3). We propose that the lack of an association between care by other helpers and lactation is due to differences in the costs and benefits of care, and the associated tradeoffs, for parents and non-breeding helpers across types of allocare. In species with a high reproductive skew within the social group, such as meerkats, breeders reduce their care levels and divert energy towards future reproduction, while non-breeding helpers show high levels of post-natal offspring care, leading to greater offspring growth and survival^51^. Conversely, when reproductive skew is limited, such as in the banded mongoose (*Mungos mungo*), parents invest more in the current litter than non-breeding helpers, which instead conserve energy to reproduce themselves in the next reproductive bout^52^. Future studies could thus investigate how reproductive skew influences the evolution of male care and female fecundity once data become available for a sufficient number of species, both with and without additional alloparents.

While previous studies do not discriminate between the specific care behaviours expressed by the male, our analysis identifies carrying and provisioning the female as the behaviours that associate specifically with a reduction in lactation time (Table 3). Carrying appears to have evolved independently two or three times among primates, as well as at least twice in carnivores and twice in rodents (Fig. 1b). The low incidence of carrying behaviours in non-primate species precludes us from testing comparatively whether a shorter lactation time is associated with the presence of male care in other orders, and should therefore be re-evaluated when more data become available. By supporting females directly through provisioning, as in some primates and canids^23^,^53^, males provide additional valuable resources that allow females to wean their offspring sooner. Thus, while the behaviours expressed by males may differ between orders, the overall relationship is the same; a shorter lactation when males care. The lack of a significant association between the duration of lactation and provisioning the offspring may thus appear surprising. However, in most mammals (including carnivores, some rodents and primates) males provision the offspring post-weaning until independence, and so this behaviour is unlikely to influence female investment in milk production^54^,^55^.

An alternative hypothesis proposes that male care and a shorter lactation are counterstrategies against infanticide by males, as the former could evolve as a defence against competing males and the latter reduces the period of vulnerability to infanticide risk for the offspring^20^,^56^. In support of this hypothesis, social monogamy and male care in primates are associated with a reduction in weaning proportion, the relative duration of lactation to the overall period of maternal investment^20^. However, Lukas and Huchard^57^ find little evidence that lactation time is shorter in species with higher infanticide risk.

Our study reveals that the previously documented increase in the frequency of breeding in species with male care^19^,^27^ is mediated by a reduction in lactation time, such that females of species with male care wean the offspring earlier and consequently reproduce again sooner. Specifically, mammals with carrying by males produce more litters in a year than species without carrying, but this association becomes non-significant when the duration of lactation—which is shorter in species with carrying—is included in the model. Therefore, by accounting for the correlated evolution between life history traits, our study identifies lactation as the specific temporal stage of reproduction during which females may energetically benefit from the help of caring males. Conversely, we find that care by other helpers is significantly associated with the frequency of breeding, but not lactation time (Table 3). Altogether these results suggest that care by the male and by other alloparents relate to female fecundity through fundamentally different mechanisms. Specifically, we suggest that male care may provide an important energetic contribution towards female reproduction during lactation, while care by other individuals is likely to be more important post-weaning and may allow females to regain body condition more quickly through mechanisms such as increased foraging time^2^.

When the duration of maternal investment (lactation and gestation) is accounted for, the number of litters produced in a year is positively associated with grooming behaviour. We suggest that producing frequent litters might require more grooming than females alone can provide to keep the offspring free of ectoparasites. Ticks, for example, can lead to high levels of infant mortality (for example, up to 50% in Chacma baboons, *Papio ursinus*) as swelling around the muzzle due to tick infestation severely limits infant suckling^58^. Therefore, frequent breeding may be the evolutionary cause for the evolution of offspring grooming by males.

Of all male care behaviours, only provisioning reproducing females is associated with larger litters. Among cooperatively breeding species, larger litter size appears to be an evolutionary prerequisite for the evolution of allocare, rather than an evolutionary consequence of it^59^. Whether this is the case for male care too or whether a larger litter results from an energetic contribution by the male towards increased female fecundity, is currently unknown. However, single species studies show that, by providing protection against predators or provisioning the offspring, males directly enhance offspring survival^39^,^60^. Moreover, our analysis demonstrates that when care by helpers and provisioning of females by the male are tested together, litter size is significantly higher only in species with provisioning by the male. Altogether our study reveals that different care behaviours allow males to gain fitness benefits via increased female fecundity, and specifically when provisioning the female and supporting her in producing larger litters, most common in carnivores, or when carrying heavy offspring and allowing females to wean the offspring faster and breed again sooner, most common in primates (Table 3). We propose that the observed differences among orders in the specific associations of life history traits with male care are likely due to how frequently and how long for each male care behaviour is expressed and the costs associated with it. Virtually nothing is currently known about the timing and energetic costs of different male care behaviours in wild mammals; quantifying how strongly costs of male care underlie the associations with life history traits revealed here across all mammals and within both the better studied orders—carnivores and primates—and the more neglected ones, will be an interesting venue for future research.

Mammals with male care or care by helpers do not have larger offspring, after accounting for the relevant duration of maternal investment. This, together with the finding that lactation time is shorter in species with biparental care, indicates that offspring grow faster postnatally in species with male care, as they reach the same size at weaning as offspring of species without it but in a shorter time. Our results support suggestions that allocare, including male care, associates with greater milk energetic output and faster offspring growth^23^. Finally, we find no evidence that long-lived species are more likely to exhibit male care behaviours. Thus, unlike increased female fecundity, a longer lifespan does not seem to help males compensate for the likely loss of additional mating opportunities associated with caring.

Most studies on the evolution of male care focus on the costs and benefits of this behaviour for the male, in relation to lost mating opportunities and increased certainty of paternity^3^,^15^. Here, we demonstrate that the evolution of male care in mammals has appreciable benefits for both males and females through increased female breeding frequency, mediated by a reduction in lactation time, and increased litter size. Our study thus reveals that male care may provide a major energetic contribution specifically during the most expensive time of female reproduction, lactation. While an increased certainty of paternity may promote the evolution of male care^2^,^3^, higher female fecundity, but not a longer lifespan, contributes to reduce the energetic and opportunity costs of caring for the males. This can also help to explain why male care evolves more easily—but not exclusively—among socially monogamous species^19^,^20^, and suggests that male care is likely under strong selection to help reduce the costs of social monogamy. As a result, the association between increased female fecundity and male care may lead to a positive evolutionary feedback between the two. Our results demonstrate that the association between male care and increased female fecundity is underappreciated and should be considered when investigating the benefits and costs of evolution of parental care by males.

## Methods

### Data collection

We first identified species for which data were already available for at least two life history traits in existing large-scale datasets (Supplementary Methods). For these species we developed protocols for data comparability and collected data on male care, care by helpers, mating system and research effort, as indicated below. The total sample size in our dataset is 529 mammals with or without male care and includes species across all major orders. However, not all life history traits are available for all species. All continuous variables are log_10_-transformed to normalize their distribution. Typically, male care behaviours and care by helpers are described in the sources but there are no quantitative measures of the amount of care provided. Thus, we code all male care behaviours, care by helpers and monogamy as binary variables, with 1 indicating the presence and 0 the absence of the trait.

We collected life history data for the following variables: female adult mass (g, *n*=467), lactation time (days, *n*=440), gestation time (days, *n*=461), weaning mass (g, *n*=262), neonatal mass (g, *n*=440), litter size (*n*=499), litters per year (*n*=433) and maximum lifespan (*n*=400). When both litters per year and interbirth interval were reported for a species, we used the former for the analysis. When only interbirth interval was available, we converted this into litters per year. We also calculated ‘post-natal body mass increase' as the difference between weaning and neonatal body mass to investigate the association between male care and offspring growth postnatally. When multiple entries were found for a life history trait, we calculated the mean.

We define male care as any care behaviour by a male towards neonates or older dependent offspring (unweaned or weaned; Supplementary Methods). Following Woodroffe and Vincent^2^, we consider the following behaviours as evidence of male care: food provisioning (separating provisioning the offspring from provisioning the female), huddling with, grooming and carrying the offspring. We investigate provisioning reproducing (pregnant or lactating) females as a form of male care because this behaviour may indirectly benefit the offspring, which could receive the additional resources that the mother has acquired. We exclude defence of the offspring from our definition of male care because this behaviour can be easily confused with general territorial behaviours^2^. Likewise, we do not consider babysitting and teaching behaviours as forms of male care because they are difficult to identify reliably across a large sample of widely different species^61^.

We extracted data from the literature on male care behaviours from a variety of primary and secondary sources for the species for which life history traits are available (Supplementary References). We find data for 65 species, of which 31 provision, 27 carry, 28 groom and 19 huddle with the offspring (Fig. 1). Note that 40 species exhibit more than one male care behaviour. In 15 out of the 31 species that provision the offspring, males also provision pregnant or lactating females. We considered only species-specific descriptions of male care and excluded any entry for the whole genus or family, as closely related species may differ in the presence of male care behaviours^54^. For example, male prairie (*Microtus ochrogaster*) and pine voles (*Microtus pinetorum*) groom and huddle with the offspring, while the closely related meadow vole (*Microtus pennsylvanicus*) shows no male care of the offspring^62^. We searched for additional information in Google Scholar and Web of Science using the keywords ‘male care', ‘paternal care' or ‘biparental care', in conjunction with the species' scientific or common name (date last accessed: 22/05/2014) for both the species already known to have male care and for all species we had life history data for (see above). Furthermore, when using secondary sources, we checked all the information from these references against the original primary source and, when the cited primary sources were not available, we performed additional searches for new references as described above. When a source reported only that male care was present in a species without details of specific male behaviours, we discarded this information as ambiguous, since we could not assess whether male care conformed to our definition. If only ambiguous information was available for a species, we excluded the species from the dataset to avoid introducing any bias. As sources generally reported only observed behaviours rather than the absence of a behaviour from the behavioural repertoire of a species, we classified species as exhibiting ‘no male care' if no mention was made of males provisioning, carrying, grooming, huddling with the offspring or provisioning reproducing females.

We define care by helpers as care towards neonates or older dependent offspring (unweaned or weaned) by any individuals other than the mother or (presumed) father of the offspring. We consider carrying, grooming, huddling with and provisioning the offspring, to be forms of care by helpers and also include allonursing in our definition. Of the 529 mammals in our dataset 92 exhibit at least one of these behaviours. Data on care by helpers were extracted from a range of secondary sources that were checked against the original source whenever possible (Supplementary Methods).

Following Lukas and Clutton-Brock^19^, we define social monogamy as an association between a single breeding pair sharing a common range or territory over more than one breeding season. Data from Lukas and Clutton-Brock^19^, who compiled the largest and most recent dataset on mammalian monogamy, were then checked against primary sources and secondary sources, for example, (ref. 54), and the *Mammalian Species* monographs of the American Society of Mammalogy (Supplementary References), to ensure that the classification of mating system was at the species level rather than genus level for every species. In fact, as for male care behaviour, closely related species within a genus may vary in mating system^54^,^63^. Of the 529 species in our dataset, 78 are socially monogamous.

Finally, data for a behavioural trait, such as male parental care, may be absent from the literature because the behaviour is not exhibited in the species, or alternatively because the species is insufficiently studied for the behaviour to have been observed. In order to control for variation in research effort among species in our dataset, we include citation count as an additional independent variable in all models. Citation count is defined as the total number of papers on a species, hence the overall research effort on that species^64^. We collected data on citation count following Nunn *et al.*'s protocol^64^ for each species in our dataset, and specifically we extracted the total number of references published on each species since 1950 as reported in Web of Science, using the species' scientific name or common name as search parameters (date last accessed: 02/11/2015).

### Statistical analysis

We use PGLS models^47^,^48^, the R package ‘caper'^65^, and a commonly used and comprehensive mammalian phylogeny^66^ that includes all mammals in our dataset, to account for species' shared ancestry and quantify the strength of phylogenetic signal in the data^47^. Caper estimates PGLS model parameters in maximum likelihood^65^ and the parameter lambda (*λ*) quantifies the magnitude of the phylogenetic signal in the model residuals^47^,^67^. *λ* can vary between 0, indicating no phylogenetic signal, and 1, indicating that similarity between species is directly proportional to the amount of shared ancestry as expected under a Brownian motion model of evolution^47^. We assess the association between a life history trait of interest, entered as the response variable, and male care, entered as the predictor variable, while also accounting for the following confounding variables in all models: other life history traits associated with the life history trait of interest^49^, social monogamy, which is evolutionary associated with male care in mammals^19^,^20^, care by helpers and research effort, measured as citation counts for a species. These PGLS models are conceptually analogous to ANCOVA models where parallel slopes with different intercepts are estimated for species with or without male care^68^, while accounting for the confounding effect of all other independent variables and phylogeny. After allometry is accounted for, mammalian life history traits fall along two independent life history axes, a ‘timing' axis of reproductive events, and an ‘output' axis that primarily captures diversity in litter size and the trade-off with offspring size^49^. Thus, we follow this framework when building our models (see Supplementary Note 1 for further details). We next use a model simplification procedure starting from ‘full' models with all predictors and progressively eliminate the least significant predictors until only significant ones remain in the simplest statistically justifiable model (‘reduced models')^50^. We assess the model fit to the data of full versus reduced models, and reduced models with and without male care, using likelihood ratio (LR) test^69^ with degrees of freedom equal to the difference in the number of predictors between two competing nested models. We carry out this procedure once when investigating the association between life history traits and male care classified as any behaviour, and once when investigating the association with all individual male care behaviours entered simultaneously as independent predictors.

Because life history traits in mammals covary along two life history axes, an ‘output' axis and a ‘timing' axis^49^, we generate variance inflation factors (VIF) to assess potential multicollinearity between all predictors in our models^46^,^69^. VIFs quantify how multicollinearity between predictors increases the variance of the model's parameters. We compute VIFs for all the independent variables in our models using non-phylogenetic generalized linear models. Including phylogeny in a statistical model generally reduces the strength of association between predictors^47^, therefore our approach is conservative as VIFs are very likely to be higher in non-phylogenetic than in phylogenetic analyses. VIF scores higher than 5 indicate problematic multicollinearity in a model, and >10 extremely problematic multicollinearity. We however find no evidence of problematic multicollinearity between predictors in any of our models as all VIFs are well below 5 (Supplementary Note 1; Supplementary Tables 1–6).

Finally, by building a PGLS model as a phylogenetic *t*-test^70^ we show that species with and without male care do not differ in research effort (Supplementary Note 1; Supplementary Table 14). All statistical tests are two-tailed with a *α*-level of significance set at 0.05.

### Data availability

The dataset used for this study is available upon request from the authors and in Dryad with the identifier DOI: doi:10.5061/dryad.j909k

## Additional information

**How to cite this article:** West, H. E. R. & Capellini, I. Male care and life history traits in mammals. *Nat. Commun.* 7:11854 doi: 10.1038/ncomms11854 (2016).

## Supplementary Material

Supplementary Information Supplementary Figure 1, Supplementary Tables 1-14, Supplementary Note 1, Supplementary Methods and Supplementary References

## Figures and Tables

**Table 1:** Reduced PGLS multi-predictor models for lactation time, litters per year and litter size with male care (any behaviour) and significant individual behaviours.

		**Variable statistics**				**Model statistics**		
**Dependent**	**Independent**	***β***	**S.E.**	***t***	***P***	**ML** ***λ***	***R***^**2**^	**Lh**
Lactation	Female body mass	0.13	0.02	6.3	<0.001	0.81	0.26	45.78
	Gestation time	0.37	0.09	4.2	<0.001			
	Male care	−0.11	0.05	−2.5	0.013			
Lactation	Female body mass	0.13	0.02	6.2	<0.001	0.82	0.27	48.19
	Gestation time	0.36	0.09	4.1	<0.001			
	Carrying	−0.17	0.07	−2.6	0.010			
	Provisioning females*	−0.21	0.11	−1.9	0.057			
Litters per year	Female body mass	−0.05	0.02	−3.0	0.003	0.90	0.22	159.13
	Lactation time	−0.15	0.04	−3.9	<0.001			
	Gestation time	−0.23	0.07	−3.3	0.001			
	Male care	0.07	0.03	2.1	0.033			
	Care by helpers	0.06	0.02	2.5	0.013			
Litters per year	Female body mass	−0.05	0.02	−2.9	0.004	0.90	0.22	159.10
	Lactation time	−0.16	0.04	−4.1	<0.001			
	Gestation time	−0.23	0.07	−3.3	0.001			
	Grooming	0.08	0.04	2.2	0.035			
	Care by helpers	0.05	0.02	2.5	0.013			
Litters per year	Female body mass	−0.10	0.02	−7.1	<0.001	0.92	0.14	143.01
	Carrying	0.10	0.05	2.1	0.035			
	Care by helpers	0.06	0.02	2.6	0.010			
Litter size	Female body mass	−0.07	0.01	−5.5	<0.001	0.95	0.16	265.61
	Provisioning females	0.26	0.05	5.1	<0.001			
	Citation count	0.05	0.01	6.2	<0.001			

For each independent variable in each model we report the parameter estimate (*β*) with standard error (S.E.), *t*-statistics and *P* value, and for each model the estimated ML *λ* value, *R*^2^ and the model log-likelihood (Lh). The total sample size for models with lactation is 390 species of which 47 have male care (14 carrying, 24 provisioning, of which 12 also provision reproducing females, 18 huddling and 23 grooming), while 80 exhibit care by helpers. The total sample size for litters per year is 370 species of which 46 exhibit male care (14 carrying, 23 provisioning, 12 of which also provision reproducing females, 18 huddling and 22 grooming), while 77 exhibit care by helpers. The total sample size for models with litter size is 448 species of which 53 species exhibit male care, with 19 carrying, 26 provisioning, 13 of which also provision reproducing females, 18 huddling and 25 grooming. Full models are reported in Supplementary Tables 1, 3 and 4.

^*^For lactation time, the comparison between a full model with provisioning females (model 2, Supplementary Table 1) and a reduced model also without this predictor approaches significance (Likelihood ratio test: LR_1_=3.66, *P*=0.056).

**Table 2:** Reduced PGLS multi-predictor models for lactation time, litters per year and litter size with significant individual behaviours within orders.

			**Variable statistics**				**Model statistics**		
**Order**	**Dependent variable**	**Independent variables**	***β***	**SE**	***t*** **value**	***P*** **value**	**ML λ**	**R**^**2**^	**Lh**
Primates	Lactation	Female body mass	0.22	0.06	3.5	0.001	0.00	0.67	10.15
		Gestation	1.21	0.33	3.7	0.001			
		Carrying	−0.23	0.08	−2.8	0.007			
Carnivores	Lactation	Female mass	0.16	0.05	3.0	0.003	0.86	0.16	4.15
		Provisioning females	−0.25	0.12	−2.1	0.039			
Primates	Litters per year	Female body mass	−0.21	0.05	−4.2	<0.001	0.87	0.49	37.16
		Carrying	0.18	0.07	2.5	0.015			
		Care by helpers	0.12	0.04	3.2	0.002			
Carnivores	Litter size	Female body mass	−0.10	0.03	−3.1	0.003	0.77	0.32	44.97
		Provisioning females	0.31	0.07	4.4	<0.001			
		Citation count	0.09	0.03	3.3	0.002			

For each independent variable in each model we report the parameter estimate (*β*) with standard error (S.E.), *t*-statistics and *P* value, and for each model the estimated ML *λ* value, *R*^2^ and the model log-likelihood (Lh). Sample sizes are as follows: for lactation time 70 primate species of which 11 exhibit carrying behaviour and 80 carnivore species of which 12 provision reproducing females; litters per year in primates includes 63 species of which 10 with carrying and 33 with care by other helpers; litter size in carnivores includes 82 species, 12 with provisioning females. Full models are reported in the Supplementary Tables 8, 10 and 11.

**Table 3:** Summary of main results for male care and care by other helpers across all mammals, carnivores, primates and rodents.

**Taxon**	**All mammals**	**Carnivores**	**Primates**	**Rodents**
Male care	Shorter lactation with carrying	Shorter lactation with provisioning females	Shorter lactation with carrying	No life history trait associated with any male care behaviour
	More litters per year with grooming (and carrying*)	Larger litters with provisioning females	(More litters per year with carrying*)	
	Larger litters with provisioning females			
Care by helpers	Longer gestationMore litters per year	Larger littersLarger neonates	More litters per year	No life history traits associated with care by helpers

^*^Mediated by a reduction in lactation time.
