# Supplementary material for: Male care and life history traits in mammals
Source: Nat Commun. 2016 Jun 14;7:11854. doi: 10.1038/ncomms11854 (PMC4911630; doi:10.1038/ncomms11854)
Supplement: Supplementary Information — Supplementary Figure 1, Supplementary Tables 1-14, Supplementary Note 1, Supplementary Methods and Supplementary References [file ncomms11854-s1.pdf]

SUPPLEMENTARY FIGURES

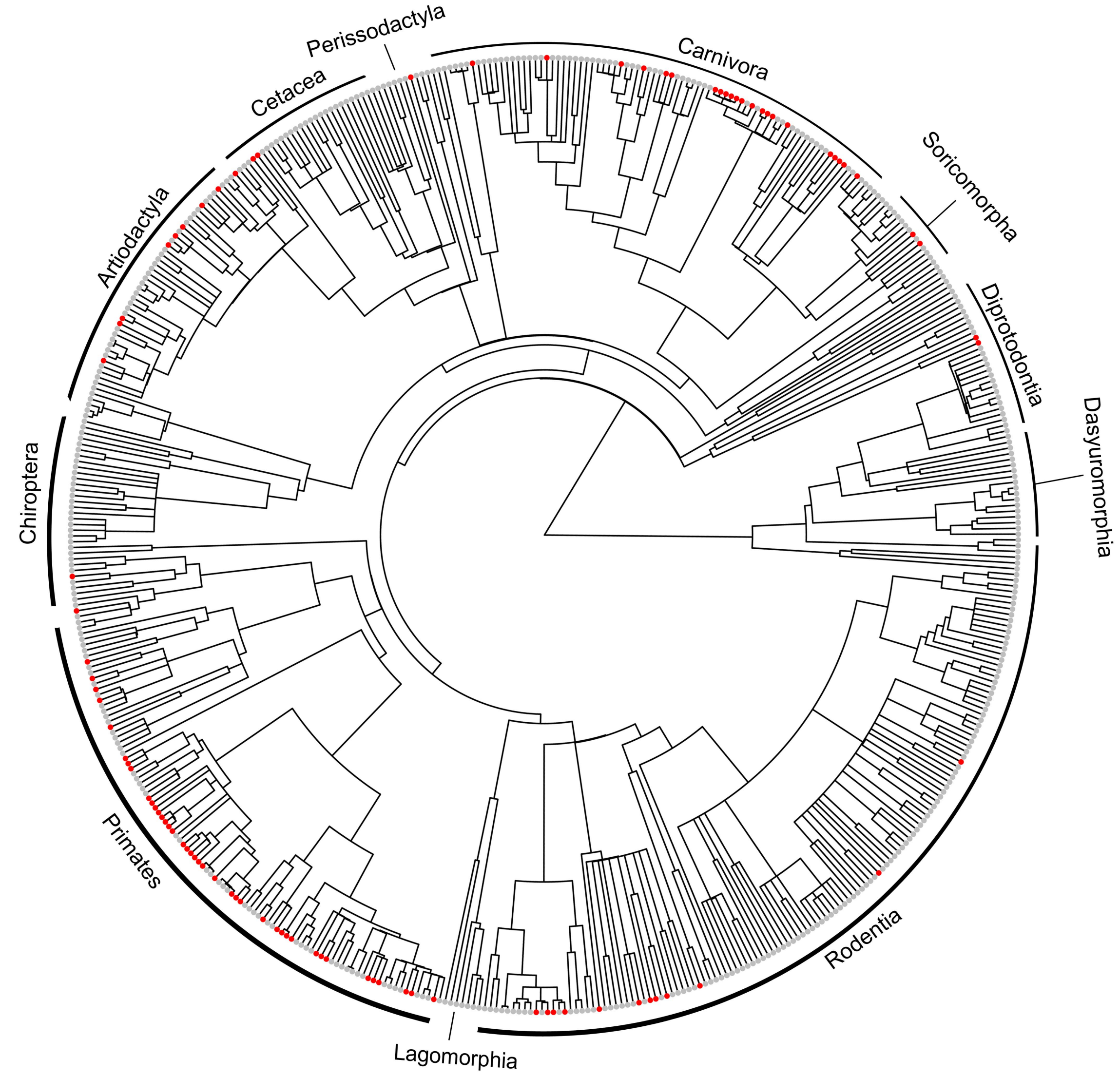

Supplementary Figure 1. Distribution of care by other helpers across the mammalian tree. Red dots indicate species with care by helpers (n=92), grey dots without care by other helpers (n=437).

SUPPLEMENTARY TABLES

Supplementary Table 1. PGLS full models for the duration of lactation for male care (model 1) and individual care behaviour (model 2). Reduced models for lactation time with male care and individual care behaviours in Table 1, main text. The sample size for these models is 390 species, of which 47 have male care, with 14 carrying, 24 provisioning, of which 12 also provision reproducing females, 18 huddling, and 23 grooming. 80 species in these models exhibit care by other helpers, while 55 are socially monogamous. Models are numbered for ease of presentation.

**Lactation time**		**Variable statistics**				**Model statistics**			
**Model n.**	**Independent variables**	**β**	**S.E.**	**t value**	**p value**	**ML λ**	**R^2**	**Lh**	**VIF**
1 (Full)	Female body mass	0.13	0.02	6.0	<0.001	0.82	0.26	46.75	2.4
	Gestation time	0.38	0.09	4.3	<0.001				2.3
	Male care	-0.11	0.05	-2.3	0.023				1.9
	Care by helpers	-0.04	0.03	-1.4	0.171				1.3
	Social monogamy	0.01	0.04	0.3	0.786				1.9
	Citation Count	0.01	0.02	0.6	0.565				1.2
2 (Full)	Female body mass	0.13	0.02	6.0	<0.001	0.82	0.27	49.49	2.5
	Gestation time	0.37	0.09	4.1	<0.001				2.4
	Carrying	-0.16	0.07	-2.3	0.025				1.4
	Provisioning females	-0.19	0.12	-1.6	0.114				2.2
	Provisioning offspring	-0.06	0.08	-0.8	0.426				2.7
	Grooming	-0.04	0.06	-0.6	0.586				2.5
	Huddling	0.01	0.07	0.2	0.849				2.4
	Care by helpers	-0.03	0.03	-1.1	0.278				1.3
	Social monogamy	0.01	0.04	0.3	0.769				1.9
	Citation count	0.01	0.02	0.4	0.701				1.2

Supplementary Table 2. PGLS full and reduced models for the duration of gestation. The reduced model is the same for both full models 1 and 3. The sample size for these models is 390 species, of which 47 have male care, with 14 carrying, 24 provisioning, of which 12 also provision reproducing females, 18 huddling, and 23 grooming. 80 species in these models exhibit care by other helpers, while 55 are socially monogamous. Models are numbered for ease of presentation.

**Gestation time**		**Variable statistics**				**Model statistics**			
**Model n.**	**Independent variables**	**β**	**S.E.**	**t value**	**p value**	**ML λ**	**R^2**	**Lh**	**VIF**
1 (Full)	Female body mass	0.10	0.01	8.8	<0.001	1.00	0.24	321.42	1.8
	Lactation time	0.07	0.02	3.1	0.002				1.7
	Male care	0.02	0.03	0.6	0.559				2.0
	Care by helpers	0.03	0.01	2.4	0.017				1.2
	Social monogamy	0.01	0.02	0.4	0.701				1.9
	Citation count	-0.01	0.01	-2.0	0.044				1.2
2 (Reduced)	Female body mass	0.10	0.01	8.8	<0.001	1.00	0.24	321.04	
	Lactation time	0.07	0.02	3.1	0.002				
	Care by helpers	0.03	0.01	2.5	0.014				
	Citation count	-0.01	0.01	-2.1	0.040				
3 (Full)	Female body mass	0.10	0.01	8.7	<0.001	1.00	0.24	321.53	1.9
	Lactation time	0.07	0.02	3.0	0.003				1.8
	Carrying	0.01	0.03	0.2	0.872				1.4
	Provisioning females	-0.01	0.04	-0.3	0.793				2.2
	Provisioning offspring	0.02	0.04	0.7	0.519				2.7
	Grooming	-0.01	0.03	-0.3	0.772				2.5
	Huddling	0.01	0.03	0.2	0.810				2.4
	Care by helpers	0.03	0.01	2.3	0.021				1.3
	Social monogamy	0.01	0.02	0.4	0.659				1.9
	Citation count	-0.01	0.01	-2.0	0.046				1.2

Supplementary Table 3. PGLS full models for the number of litters per year for male care (model 1) and individual care behaviours (models 2 & 3). Reduced models for this variable with male care and individual care behaviours in Table 1, main text. Full models 1 and 2 include the duration of maternal investment; full model 3 excludes it (see main text). The sample size is 370 species. In these models, 46 species exhibit male care, with 14 carrying, 23 provisioning, of which 12 also provision reproducing females, 18 huddling, and 22 grooming; 77 species exhibit care by helpers, while 48 are socially monogamous. Models are numbered for ease of presentation.

**Litters per year**		**Variable statistics**					**Model statistics**		
**Model n.**	**Independent variables**	**β**	**S.E.**	**t value**	**p value**	**ML λ**	**R^2**	**Lh**	**VIF**
1 (full)	Female body mass	-0.05	0.02	-2.7	0.007	0.90	0.23	159.54	2.7
	Lactation time	-0.15	0.04	-3.9	<0.001				2.3
	Gestation time	-0.24	0.07	-3.4	0.001				1.8
	Male care	0.06	0.04	1.6	0.120				2.3
	Care by helpers	0.06	0.02	2.4	0.015				1.3
	Social monogamy	0.03	0.03	0.8	0.455				2.2
	Citation count	-0.01	0.01	-0.4	0.698				1.2
2 (full)	Female body mass	-0.05	0.02	-2.6	0.009	0.90	0.23	161.43	3.0
	Lactation time	-0.15	0.04	-3.8	<0.001				2.5
	Gestation time	-0.24	0.07	-3.4	0.001				1.8
	Carrying	0.08	0.05	1.4	0.153				1.5
	Provisioning females	-0.04	0.11	-0.3	0.741				2.2
	Provisioning offspring	-0.01	0.06	-0.1	0.925				2.7
	Grooming	0.09	0.05	2.0	0.047				2.6
	Huddling	-0.07	0.06	-1.3	0.196				2.6
	Care by helpers	0.06	0.02	2.5	0.015				1.4
	Social monogamy	0.04	0.03	1.1	0.278				2.1
	Citation count	-0.00	0.01	-0.2	0.880				1.2
3 (full)	Female body mass	-0.01	0.02	-6.7	<0.001	0.92	0.15	145.80	1.2
	Carrying	0.09	0.05	1.7	0.092				1.4
	Provisioning females	0.03	0.11	0.3	0.775				2.2
	Provisioning offspring	-0.00	0.06	-0.0	0.965				2.7
	Grooming	0.10	0.05	2.0	0.047				2.5
	Huddling	-0.08	0.06	-1.3	0.186				2.4
	Care by helpers	0.05	0.02	2.1	0.035				1.3
	Social monogamy	0.04	0.04	1.0	0.310				1.9
	Citation count	0.00	0.01	0.4	0.721				1.2

Supplementary Table 4. PGLS full and reduced models for litter size for male care (model 1) and individual care behaviours (model 3). Reduced model 2 results from model simplification of full model 1; the reduced model of full model 3 with individual care behaviours is presented in Table 1, main text. The sample size for these models is 448 species. In these models, 53 species have male care, with 19 carrying, 26 provisioning, 13 of which also provision reproducing females, 18 huddling, and 25 grooming; 85 species exhibit care by helpers, while 62 are socially monogamous. Models are numbered for ease of presentation.

**Litter size**		**Variable statistics**				**Model statistics**			
**Model n.**	**Independent variables**	**β**	**S.E.**	**t value**	**p value**	**ML λ**	**R^2**	**Lh**	**VIF**
1 (full)	Female body mass	-0.07	0.01	-5.6	<0.001	0.94	0.12	254.65	1.1
	Male care	0.04	0.03	1.4	0.178				2.0
	Care by helpers	0.01	0.02	0.6	0.562				1.2
	Social monogamy	-0.03	0.03	-1.1	0.288				2.0
	Citation count	0.05	0.01	5.7	<0.001				1.2
2 (reduced)	Female body mass	-0.07	0.01	-5.7	<0.001	0.94	0.12	253.45	
	Citation count	0.05	0.01	6.3	<0.001				
3 (full)	Female body mass	-0.07	0.01	-5.5	<0.001	0.95	0.17	267.67	1.2
	Carrying	0.03	0.04	0.7	0.490				1.5
	Provisioning females	0.25	0.05	4.7	<0.001				2.0
	Provisioning offspring	0.04	0.04	0.8	0.420				2.4
	Grooming	-0.03	0.03	-1.0	0.314				2.2
	Huddling	0.00	0.04	0.1	0.923				1.9
	Care by helpers	-0.00	0.02	-0.1	0.904				1.3
	Social monogamy	-0.03	0.03	-1.2	0.218				2.1
	Citation count	0.05	0.01	5.8	<0.001				1.2

Supplementary Table 5. PGLS full and reduced models for (a) neonatal body mass and (b) body mass increase from birth to weaning for male care and individual male care behaviours. The reduced model 2 is the same for both full models 1 and 3 in both (a) and (b). The sample size in (a) is 394 species while in (b) is 232 species. In the models for neonatal mass (a), 48 species exhibit male care, with 14 carrying, 24 provisioning, 12 of which also provision reproducing females, 17 huddling, and 23 grooming; 79 species exhibit care by helpers, while 51 are socially monogamous. In the models for postnatal mass gain (b), 25 species exhibit male care, with 13 huddling, and 16 grooming; sample sizes for carrying and provisioning are too small (<10) to be used in these analyses. 49 species in (b) exhibit care by helpers, while 22 are socially monogamous. Models are numbered for ease of presentation.

**(a) Neonatal body mass**		**Variable statistics**				**Model statistics**			
**Model n.**	**Independent variables**	**β**	**S.E.**	**t value**	**p value**	**ML λ**	**R^2**	**Lh**	**VIF**
1 (full)	Female body mass	0.61	0.02	28.3	<0.001	0.97	0.81	79.27	2.4
	Gestation time	0.70	0.10	7.3	<0.001				3.3
	Litter size	-0.40	0.07	-5.4	<0.001				2.1
	Male care	0.02	0.05	0.4	0.668				2.1
	Care by helpers	0.05	0.03	1.7	0.083				1.3
	Social monogamy	-0.09	0.04	-2.3	0.024				2.0
	Citation count	-0.00	0.01	0.1	0.959				1.3
2 (reduced)	Female body mass	0.61	0.02	29.4	<0.001	0.97	0.81	77.58	
	Gestation time	0.72	0.09	7.6	<0.001				
	Litter size	-0.38	0.07	-5.3	<0.001				
	Social monogamy	-0.07	0.04	-2.1	0.037				
3 (full)	Female body mass	0.61	0.02	28.1	<0.001	0.97	0.81	80.02	2.6
	Gestation time	0.70	0.10	7.4	<0.001				3.3
	Litter size	-0.40	0.08	-5.2	<0.001				2.3
	Carrying	-0.05	0.07	-0.7	0.491				1.4
	Provisioning females	0.06	0.09	0.7	0.500				2.2
	Provisioning offspring	-0.04	0.07	-0.5	0.591				2.6
	Grooming	0.01	0.05	0.3	0.797				2.3
	Huddling	0.03	0.06	0.4	0.662				2.2
	Care by helpers	0.04	0.03	1.4	0.150				1.4
	Social monogamy	-0.08	0.04	-2.2	0.032				2.0
	Citation count	0.00	0.01	0.0	0.983				1.3

(b) Body mass increase		Variable statistics				Model statistics			
**Model n.**	**Independent variables**	**β**	**S.E.**	**t value**	**p value**	**ML λ**	**R^2**	**Lh**	**VIF**
1 (full)	Female body mass	0.83	0.02	41.6	<0.001	0.30	0.94	10.19	1.9
	Lactation time	0.19	0.06	3.2	0.002				1.9
	Litter size	-0.27	0.08	-3.2	0.002				1.8
	Male care	0.01	0.07	0.1	0.935				1.8
	Care by helpers	-0.01	0.05	-0.2	0.878				1.3
	Social monogamy	-0.07	0.07	-1.0	0.306				1.7
	Citation count	0.02	0.02	0.9	0.378				1.3
2 (reduced)	Female body mass	0.84	0.02	43.9	<0.001	0.28	0.94	9.00	
	Lactation time	0.19	0.06	3.3	0.001				
	Litter size	-0.24	0.08	-3.1	0.003				
3 (full)	Female body mass	0.83	0.02	41.1	<0.001	0.30	0.94	10.57	1.9
	Lactation time	0.17	0.06	3.2	0.002				1.9
	Litter size	-0.27	0.08	-3.2	0.002				1.8
	Grooming	0.02	0.09	0.2	0.845				2.4
	Huddling	-0.07	0.10	-0.7	0.459				2.3
	Care by helpers	-0.01	0.05	0.2	0.880				1.3
	Social monogamy	-0.05	0.07	-0.9	0.348				1.2
	Citation count	0.02	0.02	1.0	0.328				1.2

Supplementary Table 6. PGLS full and reduced models for maximum lifespan for male care and individual male care behaviours. The reduced model is the same for both full models 1 and 3. The sample size for these models is 332 species, of which 42 have male care, with 13 carrying, 22 provisioning, of which 11 also provision reproducing females, 17 huddling, and 20 grooming; 77 species in these models exhibit care by other helpers, while 45 are socially monogamous. Models are numbered for ease of presentation.

**Maximum Lifespan**		**Variable statistics**					**Model statistics**		
**Model n.**	**Independent variables**	**β**	**S.E.**	**t value**	**p value**	**ML λ**	R^2	**Lh**	**VIF**
1 (full)	Lactation time	0.12	0.04	2.8	0.005	0.86	0.26	123.49	2.13
	Gestation time	0.35	0.07	4.8	<0.001				2.01
	Litters per Year	-0.13	0.06	-2.3	0.021				2.43
	Male care	-0.02	0.04	-0.5	0.599				2.12
	Care by helpers	0.00	0.03	0.2	0.865				1.25
	Social monogamy	0.04	0.04	1.1	0.272				2.05
	Citation count	0.06	0.01	4.6	<0.001				1.11
2 (reduced)	Lactation time	0.12	0.04	2.9	0.005	0.86	0.26	122.84	2.03
	Gestation time	0.35	0.07	4.9	<0.001				1.93
	Litters per year	-0.13	0.06	-2.3	0.024				2.41
	Citation count	0.06	0.01	4.8	<0.001				1.02
3 (full)	Lactation time	0.12	0.04	2.7	0.008	0.87	0.26	125.77	2.14
	Gestation time	0.34	0.07	4.7	<0.001				2.06
	Litters per Year	-0.13	0.06	-2.2	0.029				2.53
	Carrying	-0.11	0.06	-1.7	0.085				1.50
	Provisioning females	-0.03	0.11	-0.3	0.772				2.15
	Provisioning offspring	0.03	0.06	0.5	0.597				2.72
	Grooming	0.05	0.06	0.8	0.409				2.69
	Huddling	-0.04	0.06	-0.7	0.493				2.58
	Care by helpers	0.00	0.03	0.0	0.996				1.31
	Social monogamy	0.05	0.04	1.3	0.211				2.08
	Citation count	0.06	0.01	4.7	<0.001				1.11

Supplementary Table 7. PGLS full and reduced models for lacation (a) and litters per year (b), including litter size as an additional predictor, for male care and the individual care behaviours. In both (a) and (b) reduced model 2 results from model simplification of full model 1, and reduced model 4 results from the simlification of full model 3. In (b) reduced model 6 results from the simplification of full model 5. Note that the sample sizes of these models are slightly smaller than in Table 1 and Supplementary tables 1 and 3 as there are no data on litter size for 2 species. The sample size in (a) is 389 species. In these models, 46 species have male care, with 14 carrying, 23 provisioning, 11 of which also provision reproducing females, 18 huddling, and 23 grooming. 79 species in these models exhibit care by helpers, while 49 are socially monogamous. In (b) the total sample size is 368, of which 45 species have male care, with 14 carrying, 22 provisioning, 11 of which also provision reproducing females, 18 huddling, and 22 grooming; 76 species in these models exhibit care by helpers, while 47 are socially monogamous. Models are numbered for ease of presentation.

**(a) Lactation time**		**Variable statistics**				**Model statistics**		
**Model n.**	**Independent variables**	**β**	**S.E.**	**t value**	**p value**	**ML λ**	**R^2**	**Lh**
1 (full)	Female body mass	0.12	0.02	5.8	<0.001	0.81	0.27	46.76
	Gestation time	0.34	0.09	3.6	<0.001			
	Litter size	-0.10	0.08	-1.2	0.222			
	Male care	-0.11	0.05	-2.2	0.032			
	Care by helpers	-0.04	0.03	-1.4	0.179			
	Social Monogamy	0.01	0.04	0.2	0.866			
	Citation count	0.02	0.02	0.9	0.369			
2 (reduced)	Female body mass	0.13	0.02	6.2	<0.001	0.81	0.26	44.97
	Gestation time	0.37	0.09	4.2	<0.001			
	Male care	-0.11	0.05	-2.5	0.013			
3 (full)	Female body mass	0.13	0.02	5.9	<0.001	0.82	0.28	49.13
	Gestation time	0.34	0.09	3.6	<0.001			
	Litter size	-0.07	0.08	-0.9	0.398			
	Carrying	-0.16	0.07	-2.2	0.029			
	Provisioning females	-0.17	0.12	-1.4	0.155			
	Provisioning offspring	-0.05	0.08	-0.7	0.494			
	Grooming	-0.03	0.06	-0.5	0.592			
	Huddling	0.01	0.07	0.2	0.874			
	Care by helpers	-0.04	0.03	-1.1	0.265			
	Social Monogamy	0.01	0.04	0.2	0.826			
	Citation count	0.01	0.02	0.7	0.519			
4 (reduced)	Female body mass	0.13	0.02	6.2	<0.001	0.82	0.27	47.43
	Gestation time	0.36	0.09	4.1	<0.001			
	Carrying	-0.17	0.07	-2.6	0.010			
	Provisioning females	-0.22	0.11	-2.0	0.051			

(b) Litters per Year		Variable statistics				Model statistics		
Model n.	Independent variables	β	S.E.	t value	p value	ML λ	R^2	Lh
1 (full)	Female body mass	-0.05	0.02	-2.7	0.007	0.89	0.23	158.49
	Lactation time	-0.15	0.04	-3.9	<0.001			
	Gestation time	-0.24	0.08	-3.3	0.001			
	Litter size	-0.01	0.06	-0.2	0.858			
	Male care	0.06	0.04	1.6	0.115			
	Care by helpers	0.06	0.02	2.5	0.014			
	Social Monogamy	0.03	0.03	0.7	0.473			
	Citation count	-0.01	0.01	-0.4	0.668			
2 (reduced)	Female body mass	-0.05	0.02	-3.00	0.003	0.90	0.23	158.03
	Lactation time	-0.15	0.04	-3.9	<0.001			
	Gestation time	-0.23	0.07	-3.3	0.001			
	Male care	0.07	0.03	2.2	0.033			
	Care by helpers	0.06	0.02	2.5	0.012			
3 (full)	Female body mass	-0.05	0.02	-2.6	0.001	0.90	0.23	160.29
	Lactation time	-0.15	0.04	-3.8	<0.001			
	Gestation time	-0.24	0.08	-3.3	0.001			
	Litter size	-0.00	0.07	-0.0	0.967			
	Carrying	0.08	0.05	1.4	0.157			
	Provisioning females	-0.03	0.11	-0.3	0.773			
	Provisioning offspring	-0.01	0.06	-0.1	0.933			
	Grooming	0.09	0.05	2.0	0.050			
	Huddling	-0.07	0.06	-1.3	0.204			
	Care by helpers	0.06	0.02	2.5	0.014			
	Social Monogamy	0.04	0.03	1.1	0.285			
	Citation count	-0.00	0.01	-0.2	0.830			
4 (reduced)	Female body mass	-0.05	0.02	-2.9	0.004	0.90	0.22	157.97
	Lactation time	-0.15	0.04	-4.1	<0.001			
	Gestation time	-0.23	0.07	-3.3	0.001			
	Grooming	0.07	0.04	2.2	0.035			
	Care by helpers	0.05	0.02	2.5	0.013			
5 (full)	Female body mass	-0.10	0.02	-6.3	<0.001	0.90	0.15	135.65
	Litter size	0.05	0.07	0.8	0.421			
	Carrying	0.11	0.06	1.9	0.066			
	Provisioning females	0.00	0.10	0.0	0.985			
	Provisioning offspring	-0.02	0.06	-0.4	0.704			
	Grooming	0.09	0.05	1.9	0.059			
	Huddling	-0.10	0.06	-1.6	0.104			
	Care by helpers	0.05	0.03	2.1	0.036			
	Social Monogamy	0.04	0.04	1.0	0.308			
	Citation count	-0.00	0.01	-0.2	0.808			
6 (reduced)	Female body mass	-0.11	0.01	-7.2	<0.001	0.90	0.14	132.79
	Carrying	0.11	0.05	2.2	0.032			
	Care by helpers	0.06	0.02	2.4	0.017			

Supplementary Table 8. PGLS models for the duration of lactation in primates (a), carnivores (b) and rodents (c). In (a) the sample size is 70 primate species, of which 11 exhibit male care, with 10 carrying; 35 species in these models exhibit care by other helpers, while 16 are socially monogamous. Sample sizes for provisioning, huddling, and grooming are too small (<10) to be used in these analyses. In (b) the sample size is 80 carnivore species, of which 17 have male care, all of which provision the offspring and 12 also provision the female. Sample sizes for carrying, huddling, and grooming are too small (<10) to be used in these analyses. 22 species in these models exhibit care by other helpers, while 18 are socially monogamous. In (c) the sample size is 90 rodent species, of which 18 have male care, 14 of which groom the offspring and 12 huddle with them; 16 species are socially monogamous. Sample sizes for carrying and provisioning are too small (<10) to be used in these analyses, as are the sample sizes for care by other helpers. Models are numbered for ease of presentation. The reduced models for primates (a) and carnivores (b) are in Table 2 (main text).

**(a) Primates**		**Variable statistics**				**Model statistics**		
**Model n.**	**Independent variables**	**β**	**S.E.**	**t value**	**p value**	**ML λ**	**R^2**	**Lh**
1 (full)	Female body mass	0.21	0.07	3.1	0.003	0.00	0.68	11.40
	Gestation time	1.24	0.34	3.7	0.001			
	Carrying	-0.27	0.11	-2.4	0.018			
	Care by helpers	-0.07	0.06	-1.2	0.227			
	Social monogamy	0.07	0.09	0.8	0.416			
	Citation count	0.03	0.04	0.7	0.487			

**(b) Carnivores**		**Variable statistics**				**Model statistics**		
**Model n.**	**Independent variables**	**β**	**S.E.**	**t value**	**p value**	**ML λ**	**R^2**	**Lh**
1 (full)	Female body mass	0.16	0.07	2.4	0.020	0.84	0.18	5.32
	Gestation time	-0.09	0.20	-0.4	0.660			
	Provisioning females	-0.29	0.14	-2.1	0.039			
	Provisioning offspring	-0.23	0.19	-1.2	0.233			
	Care by helpers	0.07	0.07	0.9	0.358			
	Social monogamy	0.08	0.16	0.5	0.613			
	Citation count	-0.01	0.04	-0.3	0.762			

**(c) Rodents**		**Variable statistics**				**Model statistics**		
**Model n.**	**Independent variables**	**β**	**S.E.**	**t value**	**p value**	**ML λ**	**R^2**	**Lh**
1 (full)	Female body mass	0.09	0.04	2.3	0.022	0.88	0.21	35.83
	Gestation time	0.22	0.19	1.2	0.239			
	Grooming	-0.01	0.07	-0.1	0.928			
	Huddling	-0.03	0.07	-0.4	0.725			
	Social monogamy	0.10	0.05	2.1	0.041			
	Citation count	-0.00	0.02	-0.1	0.940			
2 (reduced)	Female body mass	0.12	0.03	3.7	<0.001	0.88	0.20	34.91
	Social monogamy	0.09	0.04	2.1	0.030			

Supplementary Table 9. PGLS full and reduced models for the duration of gestation in primates (a), carnivores (b) and rodents (c). In (a) the sample size is 70 primate species, of which 11 exhibit male care, with 10 carrying offspring; 35 species exhibit care by other helpers, while 16 are socially monogamous. Sample sizes for provisioning, huddling, and grooming are too small (<10) to be used in these analyses. In (b) the sample size is 80 species, of which 17 have male care, all of which provision the offspring and 12 also provision the female. Sample sizes for carrying, huddling, and grooming are too small (<10) to be used in these analyses. 22 species in (b) exhibit care by other helpers, while 18 are socially monogamous. In (c) the sample size is 90 species, of which 18 have male care, 14 of which groom the offspring and 12 huddle with them. Sample sizes for carrying and provisioning are too small (<10) to be used in these analyses, as are sample sizes for care by other helpers. In (c) 16 species are socially monogamous. Models are numbered for ease of presentation.

**(a) Primates**		**Variable statistics**				**Model statistics**		
**Model n.**	**Independent variables**	**β**	**S.E.**	**t value**	**p value**	**ML λ**	**R^2**	**Lh**
1 (full)	Female body mass	0.07	0.02	3.7	0.001	1.00	0.34	118.06
	Lactation time	0.02	0.02	1.1	0.270			
	Carrying	-0.02	0.03	-0.6	0.552			
	Care by helpers	-0.02	0.01	-1.8	0.076			
	Social monogamy	-0.01	0.02	-0.3	0.803			
	Citation count	0.01	0.01	0.8	0.404			
2 (reduced)	Female body mass	0.09	0.02	5.0	<0.001	1.00	0.27	114.63

**(b) Carnivores**		**Variable statistics**				**Model statistics**		
**Model n.**	**Independent variables**	**β**	**S.E.**	**t value**	**p value**	**ML λ**	**R^2**	**Lh**
1 (full)	Female body mass	0.14	0.04	3.8	<0.001	1.00	0.27	53.83
	Lactation time	-0.02	0.06	-0.4	0.730			
	Provisioning females	-0.04	0.06	-0.7	0.504			
	Provisioning offspring	-0.06	0.13	-0.5	0.639			
	Care by helpers	0.06	0.03	1.8	0.073			
	Social monogamy	0.02	0.10	0.3	0.813			
	Citation count	-0.01	0.02	-0.8	0.423			
2 (reduced)	Female body mass	0.14	0.03	4.8	<0.001	1.00	0.23	51.58

**(c) Rodents**		**Variable statistics**				**Model statistics**		
**Model n.**	**Independent variables**	**β**	**S.E.**	**t value**	**p value**	**ML λ**	**R^2**	**Lh**
1 (full)	Female body mass	0.09	0.02	4.7	<0.001	1.00	0.32	94.02
	Lactation time	0.07	0.05	1.4	0.172			
	Grooming	0.01	0.03	0.3	0.744			
	Huddling	0.01	0.03	0.4	0.666			
	Social monogamy	0.02	0.02	0.8	0.420			
	Citation count	-0.02	0.01	-1.3	0.188			
2 (reduced)	Female body mass	0.10	0.02	5.4	<0.001	1.00	0.25	90.00

Supplementary Table 10. PGLS models for the number of litters per year in primates (a), carnivores (b) and rodents (c). In (a) models 1 and 2 include the duration of maternal investment, models 3 and its reduced model in Table 2 (main text) exclude it (see main text). The sample size for these models is 63 primate species, of which 11 have male care, with 10 carrying offspring; 33 species exhibit care by other helpers, while 12 are socially monogamous. Sample sizes for provisioning, huddling, and grooming are too small (<10) to be used in these analyses. In (b) the sample size is 78 carnivore species, of which 16 exhibit male care, all of which provision the offspring and 12 also provision the female. Sample sizes for carrying, huddling, and grooming are too small (<10) to be used in these analyses. In (b) 22 species exhibit care by other helpers, while 17 are socially monogamous. In (c) the sample size is 84 rodent species, of which 18 have male care, 14 of which groom the offspring and 12 huddle with them. Sample sizes for carrying and provisioning are too small (<10) to be used in these analyses, as are sample sizes for care by other helpers. In (c) 14 species are socially monogamous. Models are numbered for ease of presentation.

**(a) Primates**		**Variable statistics**				**Model statistics**		
**Model n.**	**Independent variables**	**β**	**S.E.**	**t value**	**p value**	**ML λ**	**R^2**	**Lh**
1 (full)	Female body mass	-0.19	0.06	-3.0	0.005	0.93	0.55	42.31
	Lactation time	-0.20	0.07	-3.2	0.003			
	Gestation time	0.34	0.36	1.0	0.337			
	Carrying	0.12	0.09	1.4	0.178			
	Care by helpers	0.11	0.04	2.7	0.010			
	Social monogamy	-0.01	0.07	-0.1	0.941			
	Citation count	-0.01	0.02	-0.6	0.571			
2 (reduced)	Female body mass	-0.18	0.05	-3.6	0.001	0.93	0.52	40.25
	Lactation time	-0.22	0.06	-3.7	0.001			
	Care by helpers	0.08	0.03	2.3	0.024			
3 (full)	Female body mass	-0.22	0.05	-4.0	<0.001	0.86	0.49	37.31
	Carrying	0.20	0.09	2.3	0.027			
	Care by helpers	0.12	0.04	2.9	0.005			
	Social monogamy	-0.03	0.07	-0.4	0.670			
	Citation count	-0.01	0.03	-0.3	0.755			

**(b) Carnivores**		**Variable statistics**				**Model statistics**		
**Model n.**	**Independent variables**	**β**	**S.E.**	**t value**	**p value**	**ML λ**	**R^2**	**Lh**
1 (full)	Female body mass	-0.02	0.03	-0.4	0.664	0.17	0.33	50.69
	Lactation time	-0.15	0.06	-2.6	0.013			
	Gestation time	-0.20	0.10	-2.0	0.047			
	Provisioning females	0.02	0.10	0.2	0.830			
	Provisioning offspring	0.14	0.10	1.4	0.159			
	Care by helpers	0.05	0.05	1.2	0.251			
	Social monogamy	-0.19	0.09	-2.3	0.028			
	Citation count	-0.04	0.03	-1.4	0.181			
2 (reduced)	Lactation time	-0.16	0.06	-2.8	0.006	0.22	0.24	46.44
	Gestation time	-0.24	0.07	-3.3	0.001			

**(c) Rodents**		**Variable statistics**				**Model statistics**		
**Model n.**	**Independent variables**	**β**	**S.E.**	**t value**	**p value**	**ML λ**	**R^2**	**Lh**
1 (full)	Female body mass	-0.06	0.05	-1.2	0.244	1.00	0.06	25.63
	Lactation time	0.01	0.13	0.1	0.912			
	Gestation time	-0.15	0.26	-0.6	0.571			
	Grooming	0.07	0.07	1.0	0.315			
	Huddling	-0.09	0.07	-1.3	0.216			
	Social monogamy	0.05	0.05	1.0	0.312			
	Citation count	0.01	0.03	0.2	0.852			
2 (reduced)	Female body mass	-0.06	0.04	-1.6	0.125	1.00	0.03	24.16

Supplementary Table 11. PGLS models for litter size in primates (a), carnivores (b) and rodents (c). In (a) the sample size is 84 primate species, of which 16 species have male care, with 15 carrying offspring. Sample sizes for provisioning, huddling, and grooming are too small (<10) to be used in these analyses. 40 species in (a) exhibit care by helpers, while 21 are socially monogamous. In (b) the sample size is 82 carnivore species, of which 18 have male care, all of which provision the offspring and 12 also provision the female. Sample sizes for carrying, huddling, and grooming are too small (<10) to be used in these analyses. 22 species in (b) exhibit care by other helpers, while 18 are socially monogamous. In (c) the sample size is 113 rodent species; 18 species have male care, 14 of which groom the offspring and 12 huddle with them. Sample sizes for carrying and provisioning are too small (<10) to be used in these analyses, as are sample sizes for care by other helpers. In (c) 17 species are socially monogamous. The reduced model for carnivores (b) is presented in Table 2 (main text). Models are numbered for ease of presentation.

**(a) Primates**		**Variable statistics**				**Model statistics**		
**Model n.**	**Independent variables**	**β**	**S.E.**	**t value**	**p value**	**ML λ**	R^2	**Lh**
1 (full)	Female body mass	-0.02	0.03	-0.7	0.505	1.00	0.07	103.82
	Carrying	0.01	0.04	0.2	0.847			
	Care by helpers	0.01	0.02	0.4	0.727			
	Social monogamy	0.05	0.03	1.7	0.102			
	Citation count	0.01	0.01	0.7	0.459			
2 (reduced)	Female body mass	-0.03	0.03	-1.2	0.217	1.00	0.02	101.41

**(b) Carnivores**		**Variable statistics**				**Model statistics**		
**Model n.**	**Independent variables**	**β**	**S.E.**	**t value**	**p value**	**ML λ**	R^2	**Lh**
1 (full)	Female body mass	-0.10	0.04	-2.8	0.007	0.77	0.34	45.90
	Provisioning females	0.32	0.08	3.9	<0.001			
	Provisioning offspring	0.14	0.11	1.3	0.193			
	Care by helpers	-0.01	0.04	-0.3	0.756			
	Social monogamy	-0.03	0.08	-0.4	0.698			
	Citation count	0.08	0.03	3.1	0.003			

**(c) Rodents**		**Variable statistics**				**Model statistics**		
**Model n.**	**Independent variables**	**β**	**S.E.**	**t value**	**p value**	**ML λ**	R^2	**Lh**
1 (full)	Female body mass	-0.05	0.03	-1.7	0.088	0.86	0.31	52.25
	Grooming	-0.05	0.07	-0.8	0.444			
	Huddling	0.01	0.07	0.2	0.881			
	Social monogamy	-0.09	0.04	-2.2	0.032			
	Citation count	0.11	0.02	6.3	<0.001			
2 (reduced)	Social monogamy	-0.10	0.04	-2.5	0.014	0.94	0.20	53.55
	Citation count	0.09	0.02	5.0	<0.001			

Supplementary Table 12. PGLS full and reduced models for neonatal body mass in primates (a), carnivores (b) and rodents (c). In (a) the sample size is 71 primate species, 12 species exhibit male care, with 11 carrying. Sample sizes for provisioning, huddling, and grooming are too small (<10) to be used in these analyses. In (a) 36 primate species exhibit care by helpers, while 15 are socially monogamous. In (b) the sample size is 78 carnivore species, 17 species exhibit male care, all of which provision the offspring while 12 also provision the female. Sample sizes for provisioning, huddling, and grooming are too small (<10) to be used in these analyses. In (b) 20 species exhibit care by helpers, while 16 are socially monogamous. In (c) the sample size is 90 rodent species, of which 18 species exhibit male care, 14 of which groom the offspring while 12 huddle with the offspring. Sample sizes for care by helpers in rodents are too small (<10) to be used in the analyses. In (c) 15 species are socially monogamous. Models are numbered for ease of presentation.

**(a) Primates**		**Variable statistics**				**Model statistics**		
**Model n.**	**Independent variables**	**β**	**S.E.**	**t value**	**p value**	**ML λ**	**R^2**	**Lh**
1 (full)	Female body mass	0.66	0.04	15.1	<0.001	0.90	0.89	65.07
	Gestation time	0.03	0.25	-0.1	0.922			
	Litter size	-0.53	0.15	-3.5	0.001			
	Carrying	-0.11	0.06	-1.9	0.067			
	Care by helpers	0.01	0.03	0.2	0.817			
	Social monogamy	0.05	0.05	1.0	0.344			
	Citation count	0.03	0.02	1.7	0.094			
2 (reduced)	Female body mass	0.67	0.03	19.3	<0.001	0.88	0.88	62.97
	Litter size	-0.53	0.14	-3.8	<0.001			
	Citation count	0.03	0.02	2.1	0.037			

**(b) Carnivores**		**Variable statistics**				**Model statistics**		
**Model n.**	**Independent variables**	**β**	**S.E.**	**t value**	**p value**	**ML λ**	**R^2**	**Lh**
1 (full)	Female body mass	0.57	0.07	8.7	<0.001	1.00	0.76	11.42
	Gestation time	0.30	0.21	1.4	0.159			
	Litter size	-0.62	0.16	-3.8	<0.001			
	Provisioning females	0.03	0.11	0.3	0.800			
	Provisioning offspring	-0.10	0.25	-0.4	0.685			
	Care by helpers	0.12	0.06	2.0	0.047			
	Social monogamy	-0.07	0.11	-0.7	0.506			
	Citation count	0.08	0.03	2.4	0.020			
2 (reduced)	Female body mass	0.62	0.06	10.7	<0.001	0.98	0.76	10.06
	Litter size	-0.72	0.15	-4.9	<0.001			
	Care by helpers	0.14	0.05	2.5	0.014			
	Citation count	0.08	0.04	2.2	0.034			

(c) Rodents		Variable statistics				Model statistics		
**Model n.**	**Independent variables**	**β**	**S.E.**	**t value**	**p value**	**ML λ**	**R^2**	**Lh**
1 (full)	Female body mass	0.63	0.03	19.6	<0.001	0.99	0.89	57.42
	Gestation time	0.64	0.17	3.8	<0.001			
	Litter size	-0.29	0.09	-3.2	0.002			
	Grooming	0.09	0.05	1.8	0.071			
	Huddling	-0.04	0.05	-0.8	0.436			
	Social monogamy	-0.12	0.04	-3.3	0.001			
	Citation count	-0.05	0.02	-2.3	0.027			
2 (reduced)	Female body mass	0.62	0.03	19.2	<0.001	0.98	0.89	55.47
	Gestation time	0.65	0.17	3.9	<0.001			
	Litter size	-0.30	0.09	-3.4	0.001			
	Social monogamy	-0.11	0.03	-3.2	0.002			
	Citation count	-0.04	0.02	-2.0	0.047			

Supplementary Table 13. PGLS full and reduced models for postnatal body mass gain from birth to weaning in rodents. The sample size for these models is 62 species; 15 species exhibit male care, 12 of which groom the offspring while 10 huddle with the offspring; 10 species are socially monogamous. Sample sizes for provisioning, carrying and care by helpers are too small (<10) to be used in this analysis. Models are numbered for ease of presentation.

Body mass increase		Variable statistics				Model statistics		
**Model n.**	**Independent variables**	**β**	**S.E.**	**t value**	**p value**	**ML λ**	**R^2**	**Lh**
1 (full)	Female body mass	0.77	0.05	14.0	<0.001	0.40	0.84	11.36
	Lactation time	0.21	0.18	1.2	0.241			
	Litter size	0.07	0.17	0.4	0.702			
	Grooming	0.12	0.11	1.1	0.267			
	Huddling	-0.17	0.11	-1.4	0.176			
	Social monogamy	-0.07	0.08	-0.9	0.376			
	Citation count	-0.05	0.04	-1.2	0.221			
2 (reduced)	Female body mass	0.80	0.05	17.1	<0.001	0.30	0.83	8.04

Supplementary Table 14. PGLS models for male care, social monogamy, and care by helpers, against citation count. For each independent variable in each model we report the parameter estimate (β) with standard error (SE), t-statistics and p-value, and for each model the estimated ML λ value, R^2 and the model log-likelihood (Lh). The sample size for these models is 529 species, of which 65 have male care, 92 species exhibit care by other helpers, while 78 are socially monogamous.

**Citation count**	**Variable statistics**				**Model statistics**		
**Independent Variables**	**β**	**S.E.**	**t value**	**p value**	**ML λ**	**R^2**	**Lh**
Male care	0.03	0.14	0.2	0.831	0.52	0.00	-607.40
Care by helpers	0.70	0.09	7.5	<0.001	0.59	0.10	-580.90
Social monogamy	-0.28	0.12	-2.4	0.016	0.50	0.01	-604.50

SUPPLEMENTARY NOTES

Supplementary Note 1

In Supplementary Tables 1-7 we report further details on full models for the life history traits significantly associated with male care and individual male care behaviours, and all models for the life history traits that are unrelated to male care. For each independent variable in each model we report β estimates with standard errors (S.E.), t-statistics and p-values (under 'Variable statistics'), and for each model we report the estimated lambda value (λ), R^2, model log likelihood (Lh) and the non-phylogenetic variance inflation factors (VIF, see Supplementary Methods, Statistical analysis) under 'Model statistics'. The models in each table are numbered for ease of presentation, and follow the order of presentation of results in the main text.

For each life history trait tested as a response variable, we include those life history traits known to associate with it[1] as independent variables. Thus, because life history traits in mammals covary along two independent axis, a 'timing' axis of reproductive events and an 'output' axis capturing mostly diversity in litter size and its tradeoff with neonatal body mass, we do not include litter size as a predictor in models of variables aligning along the timing axis. Including litter size in models for lactation time and litters per year confirms that this variable is not significantly associated with these variable, and its inclusions does not alter the results (Supplementary Table 7).

In Supplementary tables 8-13 we report the results of both full and reduced models in each of the three major orders - primates, carnivores and rodents - where male care behaviours are most common and so sample sizes sufficient for the analyses (i.e. the number of species with male care behaviour is equal or greater than 10). In these analyses we test individual behaviours but not male care based on all behaviours together, as diversity in male care behaviour is lower within orders than across all mammals. We test care by helpers only in models for carnivores and primates because less than 10 rodent species exhibit care by other helpers. All other independent variables – other life history traits, mating system, and citation count – are retained in all the analyses within orders. Sample sizes are however too low for within order analyses for the following life history traits, used

as dependent variables, as less than 10 species with male care behaviours are retained: maximum longevity (all orders) and body mass gain from birth to weaning (primates and carnivores).

Analyses within orders show that lactation time is significantly shorter and females produce significantly more litters per year in primates where males carry the offspring (Supplementary Tables 8 & 10). In carnivores, species in which males provision the female have significantly shorter lactation and larger litters (Supplementary Tables 8 & 11). In rodents we find no significant association between any life history traits and either grooming or huddling (Supplementary Tables 8-13).

Finally, in Supplementary Table 14 we report the results of phylogenetic t-tests[2] between citation count and male care, social monogamy, and care by helpers. We find no significant association between citation count and male care, indicating that species with and without male care do not differ in research effort. However, citation counts is higher in species with care by helpers and lower in socially monogamous species, suggesting that research effort is greater for species exhibiting care by helpers and polygynous mating system.

We conclude that our results are robust and not influenced by the correlated evolution of history traits[1], small levels of multicollinearity between predictors; differences in research effort among species; and confounding variables such as monogamy and care by helpers.

SUPPLEMENTARY METHODS

Data collection

We collected life history data from a range of available databases[1,3–9] (see also main text, Methods, Data collection). Data on male care were collected from a variety of primary and secondary sources[10–45], for species with life history data (see main text, Methods, Data collection, for data collection, data comparability protocols and sample sizes). Data for care by helpers were also

extracted from a range of secondary literature sources[17,46–49] and where possible checked against the original primary source.

We define male care as any of the following behaviours, performed by an adult male towards neonates or dependent offspring; provisioning, carrying, grooming, and huddling (see main text). We consider a form of male care behaviour the provisioning of a pregnant or lactating female by the male (see main text). We include cases where offspring are either unweaned or weaned, provided the offspring are still heavily dependent on the parents for survival. For example, wolves (*Canis lupus*) provision the pups for at least a few months post-weaning[50]; likewise in tamarinds and marmosets, helpers, including males, carry the offspring for a few more weeks post-weaning[51].

Previous studies classify as male care only provisioning or carrying the offspring, as these behaviours are believed to be more costly than huddling and grooming[19,47]. Male tamarinds (*Saguinus oedipus*) that carry their offspring lose up to 11% of body weight between birth and weaning[52], while males of other primate species have reduced foraging efficiency and face greater risk of predation when carrying the offspring[53–55]. Although less well studied, the costs of huddling and grooming, however, appear to be substantial in the few species where they have been quantified. For example, male prairie voles (*Microtus ochrogaster*) and dwarf lemurs (*Cheirogaleus medius*) that huddle with their offspring exhibit a significant reduction in body mass[29,56]. Importantly, the loss of body mass in males over the course of reproduction are more likely to be due to care behaviours, including grooming and huddling with the offspring, than the cohabitation with pregnant females[57]. Among primates grooming is associated with reduced foraging time and increased exposure to parasites[58,59]. Thus we include grooming and huddling in our definition of male care along with carrying and provisioning.

SUPPLEMENTARY REFERENCES

1. Bielby, J. Mace, G. M., Bininda-Emonds, O. R. P., Cardillo, M., Gittleman, J. L., Jones, K. E., Orme, C. D. L. & Purvis, A. The fast-slow continuum in mammalian life history: an empirical reevaluation. *Am. Nat.* **169,** 748–57 (2007).
2. Organ, C. L., Shedlock, A. M., Meade, A., Pagel, M. & Edwards, S. V. Origin of avian genome size and structure in non-avian dinosaurs. *Nature* **446,** 180–4 (2007).
3. Jones, K. E. Bielby, J., Cardillo, M., Fritz, S. A., O'Dell, J., Orme, C., David L., Safi, K., Sechrest, W., Boakes, E. H., Carbone, C., Connolly, C., Cutts, M. J., Foster, J. K., Grenyer, R., Habib, M., Plaster, C. A., Price, S. A., Rigby, E. A., Rist, J., Teacher, A., Bininda-Emonds, O. R. P., Gittleman, J. L., Mace, G. M. & Purvis, A. PanTHERIA: a species-level database of life history, ecology, and geography of extant and recently extinct mammals. *Ecology* **90,** 2648–2648 (2009).
4. Capellini, I., Venditti, C. & Barton, R. A. Placentation and maternal investment in mammals. *Am. Nat.* **177,** 86–98 (2011).
5. Silva, M. & Downing, J. A. CRC handbook of mammalian body masses. (1995).
6. Barton, R. A. & Capellini, I. Maternal investment, life histories, and the costs of brain growth in mammals. *Proc. Natl. Acad. Sci. U. S. A.* **108,** 6169–74 (2011).
7. Ernest, S. K. M. Life history characteristics of placental nonvolant mammals. *Ecology* **84,** 3402–3402 (2003).
8. Carey, J. & Judge, D. Longevity records: Life spans of mammals, birds, amphibians, reptiles, and fish. *Gerontology* **48,** 59–60 (2002).
9. Tacutu, R., Craig, T., Budovsky, A., Wuttke, D., Lehmann, G., Taranukha, D., Costa, J., Fraifeld, V. E. & de Magalhães, J. P. Human Ageing Genomic Resources: integrated databases and tools for the biology and genetics of ageing. *Nucleic Acids Res.* **41,** D1027–33 (2013).
10. Walton, L. R. & Joly, D. O. Canis mesomelas. *Mamm. Species* **715,** 1–9 (2003).
11. Audet, A. M., Robbins, C. B. & Larivière, S. Alopex lagopus. *Mamm. Species* **713,** 1–10 (2002).

12. Gompper, M. E. & Vanak, A. T. Vulpes bengalensis. *Mamm. Species* **795,** 1–5 (2006).

13. Egoscue, H. J. Vulpes velox. *Mamm. Species* 1–5 (1979).

14. Wolovich, C. Food sharing in monogamous owl monkeys (*Aotus spp*). (2006).

15. Wolovich, C. Food transfers to young and mates in wild owl monkeys (*Aotus azarai*). *Am. J. Primatol.* **70**, 211-221 (2008).

16. Rotundo, M., Fernandez-Duque, E. & Dixson, A. Infant development and parental care in free-ranging *Aotus azarai azarai* in Argentina. *Int. J. Primatol.* **26**, 1459-1473 (2005).

17. Nowak. *Walker's Mammals of the World*. (JHU Press, 1999).

18. Mason, W. & Mendoza, S. *Primate social conflict*. (Suny Press, 1993).

19. Lukas, D. & Clutton-Brock, T. H. The evolution of social monogamy in mammals. *Science* **341,** 526–30 (2013).

20. Dettling, A. Reproduction and development in Goeldi's monkey (*Callimico goeldii*). *Evol. Anthropol. Issues, News, and Reviews* **11**, 207-210 (2002).

21. Santos, C. & Martins, M. Parental care in the buffy-tufted-ear marmoset (*Callithrix aurita*) in wild and captive groups. *Rev. Bras. Biol.* (2000).

22. Wakenshaw, V. The management and husbandry of Geoffroy's marmoset. *Int. Zoo News* (1999).

23. Yamamoto, M., Box, H., Albuquerque, F. & Arruda, M. Carrying behaviour in captive and wild marmosets (*Callithrix jacchus*): A comparison between two colonies and a field site. *Primates* **37**, 297-304 (1996).

24. Nunes, S., Fite, J. & French, J. Variation in steroid hormones associated with infant care behaviour and experience in male marmosets (*Callithrix kuhlii*). *Anim. Behav.* **60**, 857-865 (2000).

25. Townsend, W. *Callithrix pygmaea*. *Mamm. Species* **665**, 1-6 (2001).

26. Oftedal, O. T. & Gittleman, J. L. in *Carnivore Behavior, Ecology, and Evolution; Volume 1* (ed. Gittleman, J. L.) pp.355–379 (Springer US, 1989).

27. Malcolm, J. R. Paternal Care in Canids. *Integr. Comp. Biol.* **25,** 853–856 (1985).

28. Sharpe, F. & Rosell, F. Time budgets and sex differences in the Eurasian beaver. *Anim. Behav.* **66**, 1059-1067 (2003).

29. Fietz, J. & Dausmann, K. H. Costs and potential benefits of parental care in the nocturnal fat-tailed dwarf lemur (*Cheirogaleus medius*). *Folia Primatol.* **74,** 246–58 (2003).

30. Tardif, S., Richter, C. & Carson, R. Effects of sibling-rearing experience on future reproductive success in two species of callitrichidae. *Am. J. Primatol.* **6**, 377-380 (1984).

31. Elwood, R. Paternal and maternal behaviour in the Mongolian gerbil. *Anim. Behav.* **23**, 766-772 (1975).

32. Hartung, T. & Dewsbury, D. Paternal behavior in six species of muroid rodents. *Behav. Neural Biol.* **26**, 466-478 (1979).

33. McGuire, B. & Henyey, E. Parental behavior at parturition in prairie voles (*Microtus ochrogaster*). *J. Mammal* **84**, 513-523 (2003).

34. Fauske, J., Andreassen, P. & Ims, R. Spatial organization in a small population of the root vole *Microtus oeconomus* in a linear habitat. *Acta Theriol. (Warsz).* (1997).

35. Oliveras, D. & Novak, M. A comparison of paternal behaviour in the meadow vole *Microtus pennsylvanicus*, the pine vole *M. pinetorum* and the prairie vole *M. ochrogaster*. *Anim. Behav.* **34,** 519–526 (1986).

36. Woodroffe, R. & Vincent, a. Mother's little helpers: Patterns of male care in mammals. *Trends Ecol. Evol.* **9,** 294–7 (1994).

37. Drygala, F., Stier, N., Zoller, H. & Mix, H. Spatial organisation and intra-specific relationship of the raccoon dog *Nyctereutes procyonoides* in Central Europe. *Wildlife Biol.* **14**, 457-466 (2008).

38. Ebensperger, L. & Ramírez-Otarola, N. Early fitness consequences and hormonal correlates of parental behaviour in the social rodent, *Octodon degus*. *Physiol. Behav.* **101**, 509-517 (2010).

39. McCarty, R. & Southwick, C. Patterns of parental care in two cricetid rodents, *Onychomys torridus* and *Peromyscus leucopus*. *Anim. Behav.* **25**, 945-948 (1977).

40. Pauw, A. Parental care in a polygynous group of bat-eared foxes, *Otocyon megalotis* (Carnivora: Canidae). *African Zool.* **35**, 139-145 (2000).

41. Dewsbury, D. Paternal behavior in rodents. *Am. Zool.* **25**, 841-852 (1985).

42. Runcie, M. Biparental care and obligate monogamy in the rock-haunting possum, *Petropseudes dahli*, from tropical Australia. *Anim. Behav.* **59**, 1001-1008 (2000).

43. Lappan, S. The effects of lactation and infant care on adult energy budgets in wild siamangs (*Symphalangus syndactylus*). *Am. J. Phys. Anthropol.* **140,** 290–301 (2009).

44. Roemer, G. & Smith, D. The behavioural ecology of the island fox (*Urocyon littoralis*). *J. Zool.* **255**, 1-14 (2001).

45. Garrott, R. Arctic fox denning behavior in northern Alaska. *Can. J. Zool.* **62**, 1636-1640 (1984).

46. Lukas, D. & Clutton-Brock, T. Life histories and the evolution of cooperative breeding in mammals. *Proc. Biol. Sci.* **279,** 4065–70 (2012).

47. Isler, K. & van Schaik, C. P. Allomaternal care, life history and brain size evolution in mammals. *J. Hum. Evol.* **63,** 52–63 (2012).

48. Snowdon, C. T. Infant care in cooperatively breeding species. *Adv. Study Behav.* **25,** 643–689 (1996).

49. Silk, J. B. The adaptive value of sociality in mammalian groups. *Philos. Trans. R. Soc. Lond. B. Biol. Sci.* **362,** 539–59 (2007).

50. Mech, D. L. Canis lupus. *Mamm. Species* **37,** 1–6 (1974).

51. Bales, K., Dietz, J., Baker, A., Miller, K. & Tardif, S. D. Effects of allocare-givers on fitness of infants and parents in callitrichid primates. *Folia Primatol.* **71,** 27–38 (2000).

52. Sánchez, S., Peláez, F., Gil-Bürmann, C. & Kaumanns, W. Costs of infant-carrying in the cotton-top tamarin (*Saguinus oedipus*). *Am. J. Primatol*. **48**, 99–111 (1999).

53. Altmann, J. & Samuels, A. Costs of maternal care: infant-carrying in baboons. *Behav. Ecol. Sociobiol.* **29,** (1992).

54. Wright, P. C. Patterns of paternal care in primates. *Int. J. Primatol.* **11,** 89–102 (1990).

55. Schradin, C. & Anzenberger, G. Costs of infant carrying in common marmosets, *Callithrix jacchus*: an experimental analysis. *Anim. Behav.* **62,** 289–295 (2001).

56. Campbell, J. C. Laugero, K. D., Van Westerhuyzen, J. A., Hostetler, C. M., Cohen, J. D. & Bales, K. L. Costs of pair-bonding and paternal care in male prairie voles (*Microtus ochrogaster*). *Physiol. Behav.* **98,** 367–373 (2009).

57. Saltzman, W., Harris, B. N., Jong, T. R., Nguyen, P. P., Cho, J. T., Hernandez, M., & Perea ‐ Rodriguez, J. P. Effects of Parental Status on Male Body Mass in the Monogamous, Biparental California Mouse. *J. Zool.* **296,** 23–29 (2015).

58. Nunn, C. L. & Altizer, S. *Infectious diseases in primates: behavior, ecology and evolution.* (Oxford University Press, 2006).

59. Moore, J. *Parasites and the behavior of animals.* (Oxford University Press, 2002).
